# Single-molecule investigations of single-chain cellulose biosynthesis

**DOI:** 10.1073/pnas.2122770119

**Published:** 2022-09-26

**Authors:** Mark A. Hilton, Harris W. Manning, Ireneusz Górniak, Sonia K. Brady, Madeline M. Johnson, Jochen Zimmer, Matthew J. Lang

**Affiliations:** ^a^Department of Chemical and Biomolecular Engineering, Vanderbilt University, Nashville, TN 37235;; ^b^Department of Molecular Physiology and Biological Physics, University of Virginia, Charlottesville, VA 22908;; ^c^HHMI, Chevy Chase, MD 20815;; ^d^Department of Molecular Physiology and Biophysics, Vanderbilt University School of Medicine, Nashville, TN 37235

**Keywords:** single-molecule studies, biosynthesis, cellulose synthase, optical tweezers, cellulose

## Abstract

Cellulose biosynthesis in sessile bacterial colonies originates in the membrane-integrated bacterial cellulose synthase (Bcs) AB complex. We utilize optical tweezers to measure single-strand cellulose biosynthesis by BcsAB from *Rhodobacter sphaeroides*. Synthesis depends on uridine diphosphate glucose, Mg^2+^, and cyclic diguanosine monophosphate, with the last displaying a retention time of ∼80 min. Below a stall force of 12.7 pN, biosynthesis is relatively insensitive to force and proceeds at a rate of one glucose addition every 2.5 s at room temperature, increasing to two additions per second at 37°. At low forces, conformational hopping is observed. Single-strand cellulose stretching unveiled a persistence length of 6.2 nm, an axial stiffness of 40.7 pN, and an ability for complexes to maintain a tight grip, with forces nearing 100 pN. Stretching experiments exhibited hysteresis, suggesting that cellulose microstructure underpinning robust biofilms begins to form during synthesis. Cellohexaose spontaneously binds to nascent single cellulose strands, impacting polymer mechanical properties and increasing BcsAB activity.

## Significance

The most abundant biopolymer, cellulose, is utilized as cellular structural scaffolding capable of harboring life from plants to bacteria. Bacteria produce cellulose in biofilms as a means of physical and chemical protection. Using optical tweezers, we directly measure cellulose polymerization from surface-tethered, nanodisc-bound bacterial cellulose synthase AB complexes. Cellulose biosynthesis is highly processive, maintains a tight grip, and is accompanied by conformational hopping, suggesting that local folding of the fiber may help transport the native strand forward. Stretching of individual cellulose strands exhibits hysteresis due to microstructure unfolding and reveals a persistence length of ∼6 nm and an axial stiffness of ∼40 pN.

Cellulose is an integral structural component utilized by several kingdoms of life for its high mechanical strength and chemical stability (1, 2). Lately, cellulose’s contribution to cell walls and microbial mats has garnered great interest as cellulosic biofuels become increasingly competitive (3) and as cellulose-stabilized bacterial biofilms are shown to play significant roles in pathogenesis (4–6). Cellulose is a polysaccharide composed of repeating glucosyl units linked by β (1–4) glycosidic bonds. Investigations of its crystalline fibrillar form show that strands are linearly arranged and flat (7). In gram-negative bacteria, cellulose is manufactured through a multisubunit transenvelope bacterial cellulose synthase (Bcs) complex containing the evolutionarily conserved (8) catalytic BcsA subunit and an inner membrane–anchored domain known as BcsB (9). The membrane-embedded BcsAB complex likely interacts with BcsC in the outer membrane to form a continuous transmembrane conduit for cellulose secretion. In vitro functional and structural studies on the purified *Rhodobacter sphaeroides* BcsAB complex revealed that it alone is sufficient for cellulose synthesis and secretion across the inner bacterial membrane (9). BcsA is allosterically activated by cyclic diguanosine monophosphate (c-d-GMP), enabling its glycosyltransferase domain to bind the Mg^2+^-coordinated uridine diphosphate glucose (UDP-glc) substrate (10, 11). UDP-glc reacts with and elongates the nonreducing terminal end of the cellulose chain one glucose unit at a time, releasing UDP by-product afterward (12). Subsequently, the polymer translocates through a transmembrane channel formed by BcsA and is likely guided into the periplasmic space by BcsB (13). Surprisingly, the degree of processive polymerization from cellulose synthases of different origins ranges from hundreds to thousands of glucose units (14, 15).

The cellulose polymer produced by BcsAB is a main component of biofilm matrices that encase sessile bacterial colonies, particularly among enterobacteria (6). Adherent bacterial populations besiege industrial systems by plugging filters, corroding metal surfaces, and fouling pipes (16). In healthcare settings, robust biofilms are responsible for ∼65% of nosocomial infections and are considerably resistant to antimicrobial treatments (5, 17). Inhibiting the production of extracellular polymeric substances, such as polysaccharides, is a strong potential antibiofilm strategy (18). Thus, a molecular understanding of bacterial cellulose synthesis is paramount for the development of powerful antibacterial agents.

BcsAB has been well described by crystallographic snapshots and in vitro analyses; however, these methods lack details of biosynthesis at the molecular level (13, 19). Extensive work has been done to characterize cellulose synthesis and the properties of cellulose (1, 2, 9, 10, 13, 19–21). Cellulose, as an abundant wall polymer of vascular plants, has been described substantially in its amorphous and crystalline forms using X-ray diffraction (22), molecular dynamics simulations (22, 23), and atomic force microscopy (24), among other methods (20, 25, 26). In all cases, studies included cellulose aggregates or atomistic models. While it is known that BcsAB produces high-molecular-weight amorphous cellulose (8), the physical and dynamic properties of single cellulose chain synthesis leading to this structure have not been characterized.

A real-time, molecular-scale analysis of cellulose synthesis and single-chain cellulose offers essential insight into the formation and structural qualities of this abundant biopolymer. Biosynthesis requires multiple elements, including activated glucose, c-d-GMP, and Mg^2+^. Furthermore, product transport and product microstructure may also impact biosynthesis. Cellulose production may be impacted by mechanical force, as seen in other molecular machines (27–29). Here, we use optical tweezers to directly probe mechanical and catalytic activity of single BcsAB molecules and their single-strand cellulose polymer products.

## Results

### Single-Molecule Activity and Biochemical Dependence of BcsAB.

The cellulose synthase BcsAB complex from *R. sphaeroides* has been successfully expressed and purified in prior studies (12, 13). The complex is catalytically active in detergent-solubilized and lipid nanodisc-reconstituted states (8), providing an ideal model system for single-molecule measurements. Accordingly, the BcsAB complex was reconstituted into MSP1D1 lipid nanodiscs formed from *Escherichia coli* total lipid extract using a His-tagged membrane scaffold protein (see *Materials and Methods*).

**Fig. 1.**
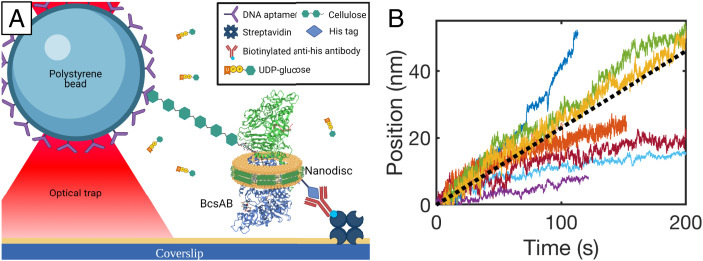
BcsAB cellulose synthesis. (*A*) Schematic of the BcsAB synthesis assay in which a single BcsAB complex (Protein Data Bank accession no. 4P00) is enveloped in a surface-bound nanodisc. A cellulose-binding DNA aptamer-coated bead binds the cellulose product strand, and the position and applied force are measured with nanometer and piconewton resolution using optical tweezers. (*B*) Cellulose synthesis traces. The black dotted line indicates the average velocity of 0.22 ± 0.01 nm s^−1^ (SEM, *n* = 176).

Cellulose synthesis was directly monitored with a tethered bead assay configuration (Fig. 1*A*). Our motility assay utilized a flow cell fabricated from a microscope slide and a KOH-etched coverslip with a gasket of double-sided sticky tape. Streptavidin, blocking protein, biotinylated anti-His antibodies, and the aforementioned nanodiscs containing BcsAB were deposited sequentially through a series of buffer exchanges and incubations. Beads decorated with cellulose-binding DNA aptamers (30) were introduced, initiating tether formation to free cellulose strands emanating from surface-bound synthases. After an incubation period to permit bead binding, a wash step removed free beads. A motility buffer containing UDP-glc, c-d-GMP, and Mg^2+^ was introduced. Tethered beads showing significant mobility after 15 min indicate actively synthesizing complexes. These beads were located by eye, centered in the measurement zone, and trapped. The sample stage was translated until the desired tension was applied to the strand. Motility traces monitoring the bead position relative to the center of the trap were recorded as described below.

Motility records were generally captured for ∼5 min, yielding synthesis trajectories ∼10 to 40 nm in length, depending on the collection window size, with some trajectories reaching as long as 100 nm. Linear fits to motility traces reveal BcsAB synthesizes cellulose at an average velocity of 0.22 ± 0.01 nm s^−1^ (SEM, *n* = 176) at 21 °C, with velocities ranging from 0.05 to 0.7 nm s^−1^. Example traces are shown in Fig. 1*B*. Force is proportional to the bead’s distance from the trap center and decreases as cellulose is synthesized. Tether lengths varied in size (400 nm to 3 μm). Motility trajectories were typically straight, maintained a constant velocity, and lacked long pauses. In some cases, abrupt extensions and retractions were observed as described below. The observed rate of synthesis in our isolated minimal system is lower than reported rates gathered through other methods, including elevated temperatures: 1.5 nm s^−1^ (31), 2 nm s^−1^ (32), and 2.5 to 9 nm s^−1^ (33) (detailed in *SI Appendix*, Table S1). Preliminary motility studies at elevated temperatures show a substantial increase in activity, with a mean velocity of 1.2 ± 0.1 nm s^−1^ (SEM, *n* = 50) at 37 °C, consistent with the literature (*SI Appendix*, Fig. S1). Additionally, components absent from our single-molecule studies, such as BcsC, could further enhance the cellulose synthesis rates (31).

**Fig. 2.**
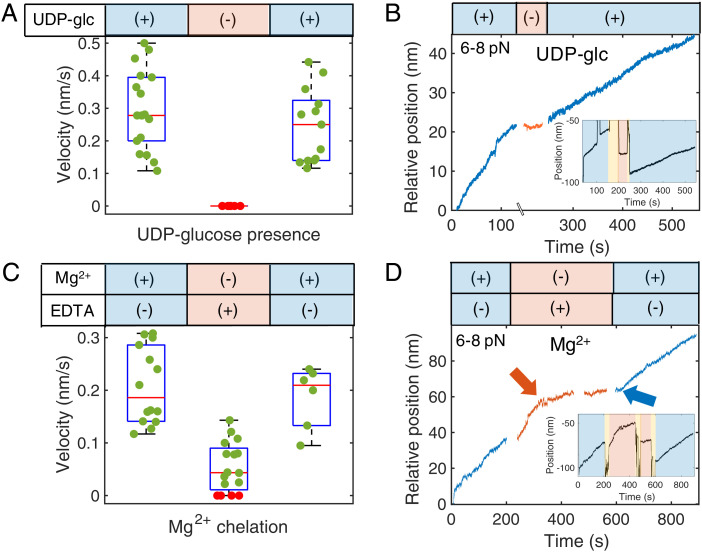
Biochemical controls. (*A*) All BcsAB complexes sampled in the absence of UDP-glc (*n* = 10) showed no activity, while synthases sampled before removal (*n* = 18) and after replenishment (*n* = 13) displayed clear motility. Green and red points indicate synthesis and no synthesis of individual BcsAB, respectively. Distributions are shown as box plots. (*B*) An example trace of UDP-control buffer exchanges while monitoring the same tether shows that synthesis halts without available monomer. (*C*) Sampled synthases before introduction (*n* = 14), in the presence (*n* = 16), and after removal (*n* = 6) of an EDTA control buffer reveal that velocity slows to 0.05 ± 0.01 nm s^−1^ (SEM). Distributions are shown as box plots. (*D*) Mg^2+^/EDTA control buffer exchange on a single tether indicates synthesis is severely hindered after Mg^2+^ chelation. Red and blue arrows point to the moments Mg^2+^ was chelated and replenished, respectively. All activity was recovered when both control buffers were washed out. *Insets* in *B* and *D* are the raw traces, including large perturbations where buffers were exchanged midexperiment. Blue indicates a complete synthesis solution, yellow indicates flow, and red indicates control buffer. We note the force range for each example trace, with the typical range spanning 2 to 3 pN. The break between control regions in *D* at ∼450 s is from preparing the next flow step, but no buffer exchange occurred.

To confirm that these records depend on synthase activity, we investigated the effects of critical assay components such as UDP-glc and Mg^2+^ on catalysis by probing for activity under varying control conditions. In general, we randomly sampled multiple synthases before and after washing out the synthesis buffer and replacing it with three flow channel volumes (3 × 15 μL) of control buffer. We sampled again after replenishing the system with the synthesis buffer. In some experiments, we were able to monitor continued activity of the same tether. All controls were sampled between 3 and 8 pN of applied force, with a mean of 6 pN. Force nominally changes within a finite range of 2 to 3 pN along a given trajectory, but rates were unaffected by force within this range. As expected, the removal of the substrate UDP-glc suspended cellulose production in all cases until the fuel was reintroduced, at which point BcsAB resumed normal catalysis (Fig. 2 *A* and *B*). To confirm Mg^2+^ dependence, 50 mM ethylenediaminetetraacetic acid (EDTA) was included in the control buffer, in addition to excluding Mg^2+^, to chelate any residual ions. Sampled complexes in EDTA showed a 75% decrease in synthesis velocity (0.05 ± 0.01 nm s^−1^, SEM) from those sampled before chelation (Fig. 2 *C* and *D*), and again, velocity was rescued with reintroduction. Thus, as expected, Mg^2+^ facilitates catalysis (10).

### C-d-GMP Refuses Dissociation.

C-d-GMP is an allosteric activator of BcsA and binds to its C-terminal PilZ domain. Binding of c-d-GMP mobilizes a gating loop necessary for substrate binding to BcsA’s catalytic pocket (10). It was previously unclear whether the activator remains bound to BcsA during cellulose biosynthesis or is released from the enzyme after the initiation reaction.

**Fig. 3.**
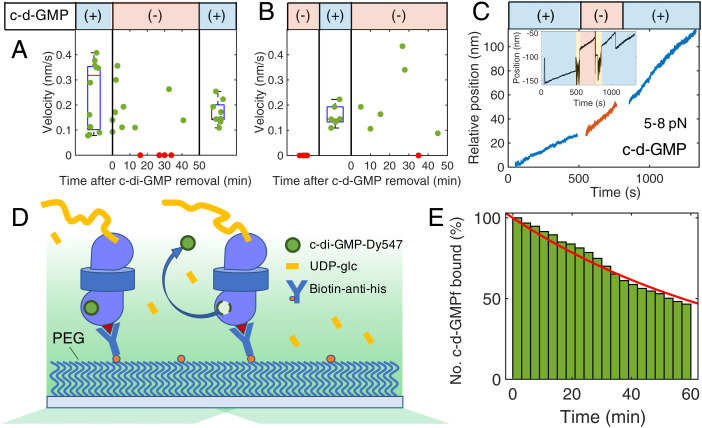
C-d-GMP controls and fluorescence. (*A*) Different complexes were sampled for synthesis before removal, at varying time points after removal, and after replenishment of c-d-GMP. Motility persists in most cases. (*B*) We performed a null experiment starting without c-d-GMP, introducing it to the system, and removing again. C-d-GMP is necessary for synthesis but remains bound for long periods of time. Distributions in A and B are shown as box plots. (*C*) Single-tether c-d-GMP controls show synthesis is relatively unaffected when the activator is removed from solution. The applied force range is 5 to 8 pN for this example. (*D*) Schematic of fluorescence assay in which BcsAB is bound to the surface in the same design as the synthesis experiments, except the coverslip includes a nonstick PEG brush layer between the coverslip and the complex. If c-d-GMP–labeled DY-547 (c-d-GMPf) is bound to BcsAB, we detect fluorescence. The signal disappears in a single step when the molecule dissociates or photobleaches. (*E*) The number of bound and fluorescing c-d-GMPf decreases over time (*n* = 247). We record incredibly long bond lifetimes of c-d-GMPf, as 70% of molecules remain associated past 30 min, with 46% persisting until the 60-min acquisition time limit. An exponential fit (red) reveals a time constant of 82.5 min corresponding to an off-rate of 2.0 × 10^−4^ s^−1^. Due to potential photobleaching, our results show the lower bound of the time constant.

We performed buffer exchange sampling experiments with c-d-GMP identical to those described above to identify the effects on synthesis (Fig. 3*A*). Prior to removal, all BcsAB synthases were shown to be active (*n* = 12). After removal, 71% of the synthases sampled (10 of 14) were active, with some active synthases still present at 45 min, indicating c-d-GMP binds very strongly to BcsA during synthesis and is likely required to remain bound. Stalled complexes were first detected ∼20 min after the buffer exchange. To demonstrate that c-d-GMP is necessary for motility, the buffer order was reversed, starting with a motility buffer lacking c-d-GMP (Fig. 3*B*). As expected, BcsAB initially displayed no synthesis in the buffer lacking c-d-GMP (*n* = 6). Immediately after addition of c-d-GMP, 100% of complexes sampled (*n* = 6) demonstrated production. C-d-GMP was removed again after 45 min, and the sampled synthases behaved as observed before, exhibiting catalysis with only one of seven stalling. Individual tether tests showed no immediate effect on polymerization rates (*n* = 2) after c-d-GMP depletion (Fig. 3*C*).

With apparent c-d-GMP retention times of ∼20 min or more, we developed a single-molecule total internal reflection fluorescence (TIRF) assay to directly monitor the presence of dye-labeled c-d-GMP over extended periods (Fig. 3*D*). In these studies, we used a c-d-GMP molecule with a DY-547 dye labeled to one ribose group (c-d-GMPf). Both bulk synthesis and single-molecule synthesis tests showed no change in activity in the presence of 30 μM c-d-GMPf versus unlabeled dinucleotide (*SI Appendix*, Figs. S2 and S3). BcsA binds an intercalated c-d-GMP dimer (10). In our assays, the BcsAB complexes were incubated, along with other synthesis components, with c-d-GMPf at 300 nM and unlabeled c-d-GMP at 29.7 μM to ensure the formation of mixed c-d-GMP dimers containing only a single c-d-GMPf. Just before data acquisition, c-d-GMPf was washed out with 10 times the flow cell volume (10 × 20 μL) of normal synthesis buffer so that the only remaining fluorophores were bound to BcsAB. We recorded the signal for 60 min, sampling at 0.33 s^−1^. To minimize photobleaching, images were acquired by triggering excitation for only 100 ms during each acquisition, with 120 s of total illumination. Example measurements are shown in *SI Appendix*, Fig. S4.

Consistent with single-tether measurements, 70% (172 of 246) of the c-d-GMPf remained bound for 30 min, with a considerable 46% of events (114 of 246) showing bond lifetimes longer than the 60-min acquisition limit (Fig. 3*E*). An exponential fit to the percentage of remaining c-d-GMPf over time (Fig. 3*E*) reveals a bound time constant of 82.5 min and a dissociation rate of 2.0 × 10^−4^ s^−1^. Our results represent the lower bound because of potential photobleaching. Control flow cells lacking BcsAB showed no decoration, indicating the signal from c-d-GMPf only occurs when bound to BcsAB (*SI Appendix*, Fig. S5). Once bound, most c-d-GMP refused to dissociate or exchange with others in solution.

### Energetic and Kinetic Analysis.

**Fig. 4.**
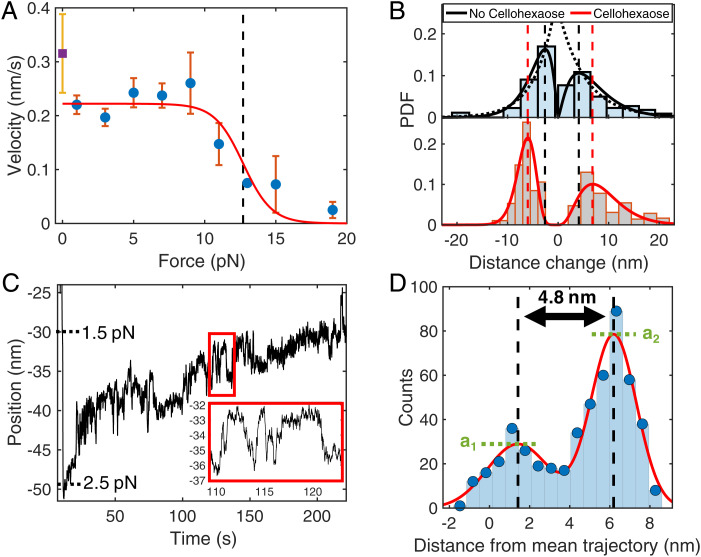
Kinetics analysis. (*A*) Recorded polymerization velocities from motility traces are binned and averaged every 2 pN (blue circles) and fit to a general Boltzmann relationship (*n* = 176), revealing a distance to the mechanical transition state of 4.0 nm. The correlation indicates synthesis is biochemically limited, and synthesis begins to stall with an assisting load of 12.7 pN (black dashed line). Unloaded velocity (purple square) was recorded from a change in contour length over time (*SI Appendix*, Fig. S9). Error bars denote SEM. (*B*) Probabilty density function (PDF) vs. extension and retraction. (*B*, *Top*) We detected a large range in extensions (2 to 100 nm, positive distance change) and retractions (2 to 20 nm, negative distance change). The average extension during motility was 10.6 ± 1.9 nm (SEM, *n* = 73), and the average retraction was −4.6 ± 0.7 nm (SEM, *n* = 26). Exponential fits (dotted black lines) generated length scales of 5.6 and −4.0 nm, respectively, while gamma distribution fits reveal peaks at 4.1 and −2.6 nm (dashed vertical black lines). (*B*, *Bottom*) In the presence of cellohexaose, the extension and retraction profiles were best fit to gamma distributions, with peak locations appearing larger than for single cellulose at 6.8 and −6.0 nm (dashed vertical red lines). Transition magnitudes below 3 nm were not observed with cellohexaose present. Outlier extensions greater than 40 nm were excluded from diagrams but included in mean calculations. (*C*) Rapid extensions and retractions of 3 to 10 nm during cellulose synthesis at ∼2 pN. Force reference markers note the slight decrease in applied force as cellulose is synthesized. (*D*) A histogram of distances from the mean trajectory for the *Inset* in *C* is fit to the sum of two Gaussian distributions separated by a displacement of 4.8 nm. The mean distance between states is 5.0 ± 0.1 nm (SEM, *n* = 201 from 51 molecules). The ratio of amplitudes (a_2_/a_1_) is equal to the ratio of the equilibrium force (1.8 ± 0.2 pN, SEM, *n* = 201 segments from 51 molecules) to the acquisition force. Very few segments (<1%) displayed multimodal behavior and were excluded from this analysis.

To determine the impact of force on synthesis rates, we constructed a force-velocity plot ranging from 2 to 20 pN (Fig. 4*A*). A fit to the general Boltzmann distribution revealed that velocity remained constant as applied force increased until a stall force of 12.7 pN, after which a decrease in activity is observed (Fig. 4*A*) (28). The fit parameters of the relationship reveal that most of the enzymatic cycle does not involve load-dependent steps. Thus, cellulose synthesis is a biochemically limited process, and force (for example, translocation) has a negligible impact on synthesis rates until the ∼13 pN level is reached, after which synthesis halts. The fits also reveal a characteristic distance of 4 nm for the load-dependent mechanical transition, which is comparable to the length of the BcsAB complex’s transmembrane channel (13). The characteristic distance represents the distance along the reaction coordinate to the transition state, the apex of the energy landscape, of a mechanical step within one full catalytic cycle.

Abrupt extension and retraction transitions ranging from 2 to 100 nm in size were frequently observed (seen in 49% of traces, Fig. 4*B* and *SI Appendix*, Fig. S6). Such features are larger than expected from incorporation of individual glucose molecules (0.56 nm) (34), and several are larger than the size of the complex (15 nm) (13). Results also revealed larger position fluctuations than those typically observed in similar tethered bead experiments (35–37). Larger extensions generally appeared earlier in traces, with few events occurring after initial extensions. To investigate the fluctuations, we analyzed 51 traces by first subtracting the average velocity and then plotting the distribution of the bead position from the mean trajectory over successive 5-s time windows. While some segments exhibited fluctuations consistent with Brownian motion, many showed the structure deviating from a single distribution, suggesting there are underlying hops in length (Fig. 4 *C* and *D*). Tests using a DNA strand of similar length and tension revealed distributions (*n* = 16) that fit well to a single Gaussian distribution (*SI Appendix*, Fig. S7). The discrete fluctuations in the cellulose strand likely originate from nonuniform motions in the synthesis machinery and/or rearrangements of the cellulose strand configuration during strand growth. Analysis of abrupt extensions and retractions during synthesis revealed exponential distributions in distance for both extension and retraction. The mean extension and retraction were 10.6 ± 1.9 nm (SEM, *n* = 73, Fig. 4*B*) and 4.6 ± 0.7 nm (SEM, *n* = 26, Fig. 4*B*), respectively. Exponential fits to the distributions of extension and retraction distances (Fig. 4*B*) yielded exponential fit lengths of 5.6 and 4.0 nm, respectively.

In some cases, when tension was held at ∼2 pN, the extensions and retractions alternated rapidly, indicating an equilibrium point and giving rise to the previously observed non-Gaussian position fluctuations (Fig. 4*C*). Interestingly, the rapid extensions and retractions were the same or very close in size for each individual molecule but varied in size between cellulose strands. Additional example traces can be seen in *SI Appendix*, Fig. S8. The mean magnitude of the fluctuations was 5.0 ± 0.1 nm (SEM, *n* = 201 over 51 traces), with >95% of events between 2.3 and 9 nm (Fig. 4*D* and *SI Appendix*, Fig. S9). Force applied to the system alters the probability that the system exists in an extended or retracted state. Higher force favors an extended state, while lower force favors a retracted one. An analysis of the ratios between Gaussian amplitudes from multiple two-state segments revealed an equilibrium force, the force applied at which both states are equally likely, of 1.8 ± 0.2 pN (SEM, *n* = 201 over 51 traces).

Additionally, the rates of extension and retraction derived from the dwell times within each state change exponentially with force. *SI Appendix*, Fig. S10 shows a schematic of such hopping between states, and *SI Appendix*, Fig. S11 contains example analyses. Calculating the kinetics as a function of force from the time domain, assuming a linear relationship between the log rate and force, yielded an equilibrium force of 2.4 pN, where the transition rate was 4 s^−1^ (*SI Appendix*, Fig. S10, *n* = 322 over 14 traces). For this analysis, only molecules exhibiting a consistent hopping distance in the range of 4 to 6 nm, representing >85% of events, were included. The bimodal nature of the traces suggest that the structural changes occur at the same site along the polymer. Given the ∼4- to 5-nm transitions occur at 2 to 3 pN of tension, the work done during these transitions is ∼8 to 15 pN·nm, which is similar to the energy of one to three hydrogen bonds (38). Because the sizes of extensions varied widely with applied force and are larger than glucose or the complex, the extensions and retractions most likely occur when the secondary structure of single-stranded cellulose unfolds or assembles microstructures via intrastrand hydrogen bonds. One explanation of the observed reversible transitions is that a hairpin-like structure of cellulose is continually opening and reforming. Alternatively, although less likely, the transitions may be constructs of the synthase’s conformational changes during synthesis. Unlike the well-defined chemical structures of DNA and RNA hairpins that have explicit distances between states (39), identical units along the cellulose strand permit varying folding sizes at multiple sites along the strand. Given the distribution seen in cellulose-based, hairpin-like transition distances (*SI Appendix*, Fig. S9), estimates from this type of analysis should only be interpreted locally.

The relative straightness or wandering of a trajectory is also an indicator of the underlying kinetics. Fewer rate-limiting steps within the catalytic cycle will lead to a more random and less consistent path of synthesis, straying from the mean trajectory. In contrast, more complex schemes, including multiple parallel steps, similar rate-limiting steps, and paths that deviate from the motility cycle, can yield a straighter motility trajectory. The rate-limiting steps within the catalytic reaction cycle are the individual chemical reactions or physical motions that determine the amount of time required to initiate, execute, and restart the cyclic biosynthesis progression unique to the processive behavior of BcsAB. An analysis of the randomness, or variance of the bead position from the mean trajectory, reveals how many similar rate-limiting steps underlie a motility cycle for a given characteristic step size (40–42). Two randomness parameters, dimensionless values of the fluctuations in polymerization cycle completion times, were calculated using two different, plausible physical step sizes of biosynthesis (*SI Appendix*, Fig. S12). We calculate randomness parameters for each individual molecule to isolate the stochasticity in the catalytic cycle from the heterogeneity in enzyme rates. When using the distance to the transition state along the reaction coordinate, given from the force-velocity fit (Fig. 4*A*), as the step size (4 nm), the mean randomness variable was 0.94 ± 0.12 (SEM, *n* = 50), indicating only one rate-limiting step. However, the complex is known to extrude cellulose one glucosyl unit at a time (12). Using the known spacing of one glucose molecule (0.56 nm) (34) yields a mean randomness parameter of 6.75 ± 0.84 (SEM, *n* = 50), compelling a model that includes off-pathway or multiple kinetic schemes (43).

### Cellulose Stretching Reveals Microstructure and Elasticity.

To investigate the extensions and larger position fluctuations observed in motility measurements and to mechanically characterize the polymer, we created a cellulose stretching assay similar to previously developed stretching assays (44–49). Single-strand cellulose synthesis by BcsAB provides an opportunity to measure the core properties of an isolated polymer. Beads tethered via a single cellulose strand were centered directly over the coverslip attachment point and pulled parallel to the surface. As force is applied, the bead is pulled out of the trap center. Strands were stretched and relaxed repeatedly.

In one set of experiments designed to track strand growth, we applied the cellulose stretching technique at various time points throughout a ∼4-min window of active synthesis to monitor velocity. By analyzing the relative apparent contour length (the tether length mapping to the polymer backbone distance) for each of the stretching time points, we were able to obtain a velocity for direct comparison to the assay that directly monitors tether synthesis (*SI Appendix*, Fig. S13). Such stretching measurements, which are nominally at zero load, revealed an average velocity of 0.32 ± 0.07 nm s^−1^ (SEM, *n* = 6), consistent with the direct monitoring of contour length under tension. In all stretching experiments, the true extension is determined from the angle of incidence of the tether (50).

**Fig. 5.**
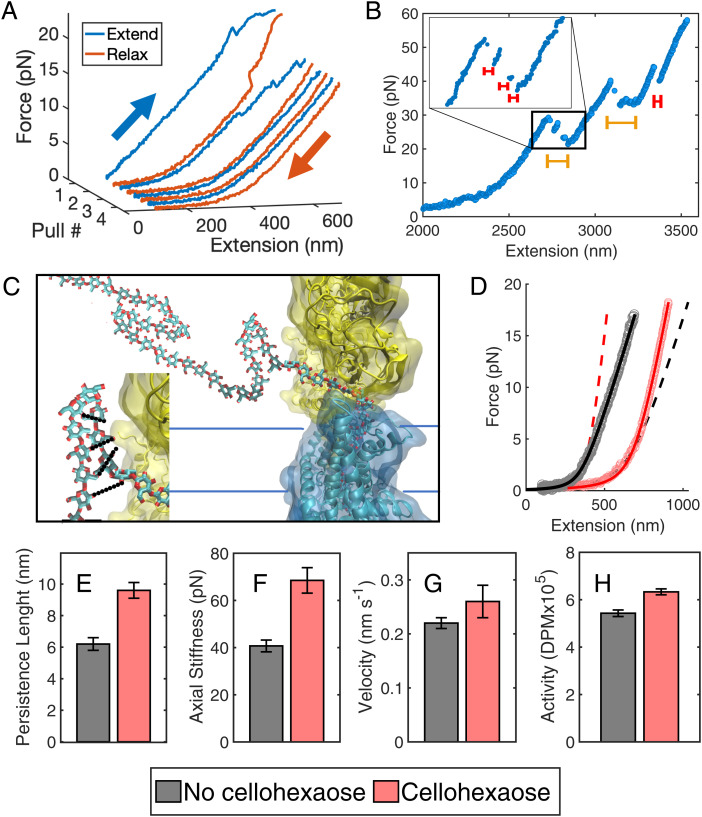
Single cellulose strand stretching and effects of cellohexaose hybridization. (*A*) At the beginning of consecutive stretches, 26% of cellulose strands exhibit hysteresis until microstructures are unfurled and cellulose reaches a fundamental state. (*B*) During stretching, cellulose undergoes sudden elongations of random distances between regions of stability. Within larger events (yellow bars) exist smaller jumps (*Inset*, red bars). The change in extension is likely due to the unfolding of microstructures. (*C*) Cellulose product likely forms hairpins or other secondary microstructures upon or after extrusion from the complex. BcsA is shown in cyan, BcsB is shown in yellow, and the residues comprising the complex’s exit pore are shown in red. Formation of the secondary structure through hydrogen bonding (*Inset*, black dotted lines) between strand segments may also assist with translocating cellulose through the synthase. (*D*) Single cellulose chain follows the eWLC model after being fully extended (gray points and black fit). Experiments with cellohexaose (pink points and red fit) show a larger persistence length and axial stiffness. The dashed lines are theoretical fits using the same contour length of each respective data curve but the persistence length and axial stiffness of the opposite condition, with or without cellohexaose. The juxtaposition highlights the change in axial stiffness caused by cellohexaose hybridization. Bar graphs display the increase in (*E*) persistence length, (*F*) axial stiffness, (*G*) single-molecule synthesis velocity, and (*H*) bulk BcsAB overnight activity, represented as disintegrations per minute (DPM), in the presence of cellohexaose. Error bars in *E*-*H* denote SEM.

In another set of stretching experiments, we allowed a strand to grow and then performed repeated stretching measurements to measure the polymer properties. Our measurements include a range of tether lengths depending on the time allowed for synthesis and the productivity of individual synthases. Hysteresis was observed, especially earlier in stretching measurements (Fig. 5*A*). In every case, hysteresis vanished after multiple successive stretches, revealing the fundamental state of single-chain cellulose. During stretching measurements, we observed abrupt extensions similar to those from motility traces in 26% of stretched cellulose strands (Fig. 5*B*).

Larger extensions appeared earlier in repeated stretching experiments before reaching a tempered state. The extensions were measured by noting the change in contour length in stretching experiments (mean: 18.1 ± 4.9 nm, SEM, *n* = 22). At forces below 5 pN, retraction/refolding events occurred, implying that a folded state may be more favorable, even under light tension. We expect a larger spread in extensions and fewer retraction events from stretching experiments compared to polymer synthesis trajectories, because the applied tension is greater and can catastrophically open multiple extension elements in one event. Both stretching and polymer synthesis traces revealed a large range of transition distances (2 to 110 nm), suggesting the presence of inhomogeneous microstructure due to cellulose folding back on itself. Models of expected microstructures, such as overhang folds or hairpins, giving rise to extensions, retractions, or hopping, are shown in Fig. 5*C*.

After repeated stretching on a single chain, cellulose resembled classical tethered polymer profiles with a low force region typical of entropic configuration rearrangement and a higher force region typical of enthalpic polymer stretching. This shape is consistent with models where the persistence length (the distance the polymer points in the same direction) is smaller than the contour length (44, 47, 49, 51). The resulting force versus extension plots were fit to the extendible worm-like chain (eWLC) model first proposed by Odijk (51) in 1995, which includes an elasticity stiffness term for the enthalpic region (Fig. 5*D*). Single cellulose chains displayed a persistence length of 6.2 ± 0.4 nm (SEM, *n* = 104) and an elasticity stiffness of 40.7 ± 2.5 pN (SEM, *n* = 78) (*SI Appendix*, Fig. S14).

### Cello-oligosaccharides Bind Nascent Cellulose Strands and Increase Activity.

With the observation of microstructure formation through self-association, we added cello-oligosaccharides (cello-oligos) to the solution to investigate their ability to bind cellulose and influence strand synthesis and mechanical properties. Here, we investigated two partially soluble cello-oligos, cellotetraose and cellohexaose. In the presence of 5 mM cellotetraose, we observed no significant change in mechanical parameters (*SI Appendix*, Table S2). However, at 50 mM cellotetraose, both cellulose’s persistence length and axial stiffness increased to 9.5 ± 0.4 nm (SEM, *n* = 113, *SI Appendix*, Table S2) and 60.9 ± 3.8 pN (SEM, *n* = 107, *SI Appendix*, Table S2), respectively. Similar changes to strand properties were observed in the presence of 0.45 mM cellohexaose, yielding a persistence length of 9.6 ± 0.5 nm (SEM, *n* = 134, Fig. 5 *D* and *E* and *SI Appendix*, Table S2) and an increase in axial stiffness to 68.5 ± 5.4 pN (SEM, *n* = 134, Fig. 5 *D*–*F* and *SI Appendix*, Table S2). The increases in persistence length and mechanical resistance to tension indicate that the cello-oligos bind and alter the mechanical properties of the nascent cellulose strand.

Microstructure formation was also impacted through hybridization of cellotetraose and cellohexaose. In cellulose–cello-oligo stretching experiments with cellohexaose, microstructure appeared in 94% of traces compared to in 26% without cello-oligos (*SI Appendix*, Table S2). In fact, cellohexaose appeared to cause frequent refolding between subsequent stretches, preventing ultimate formation of a final, homogeneous tempered state (*SI Appendix*, Fig. S15). Note that single cellulose strands in the absence of of cello-oligos will persist in this final state for a period of time, even if they are not under tension (Fig. 5*A*). Microstructure also appeared in 84% of motility experiment traces with cellohexaose compared to 49% without. Example traces are displayed in *SI Appendix*, Fig. S16. The mean extension and retraction event sizes observed in motility traces increased from 10.6 ± 1.9 nm and 4.6 ± 0.7 nm, respectively, to 13.5 ± 1.0 nm (SEM, *n* = 196) and 6.6 ± 0.3 nm (SEM, *n* = 63), respectively, in the presence of cellohexaose (Fig. 4*B* and *SI Appendix*, Table S2). There is also a clear nonzero maximum in the distribution of microstructure size (Fig. 4*B*). We also observed rapid position fluctuations in the range of 4 to 6 pN (*SI Appendix*, Fig. S11). Cellohexaose increased the frequency and size of cellulose microstructure.

Motility experiments in the presence of cellohexaose did not impede synthesis, revealing a modest increase in mean biosynthesis velocity to 0.26 ± 0.3 nm s^−1^ (SEM, *n* = 45, *P* = 0.026) from 0.22 ± 0.2 nm s^−1^ (Fig. 5*G* and *SI Appendix*, Table S2). Consistent with our single-molecule experiments, bulk activity assays of BcsAB in the presence of cellohexaose showed slightly increased cellulose biosynthesis as well (Fig. 5*H* and *SI Appendix*, Fig. S17). In bulk assays, cellulose polymers produced by different BcsAB complexes may already interact, thereby mimicking the effect observed for cellohexaose in single-molecule assays. Thus, binding of hydrophobic cellohexaose to the cellulose product not only impacts mechanical properties but increases BcsAB’s productivity.

## Discussion

Optical tweezer records provide insight into force dependence of synthesis and the ability to directly track the progress in real time. Below an assisting load of ∼13 pN, BcsAB was relatively immune to force (Fig. 4*A*). However, above ∼13 pN of tension, slowing or stalling occurred. Curiously, cellulose stretching measurements show that BcsAB maintains a tight grip to cellulose, as up to 100 pN of pulling force failed to dislodge the strand from the synthase’s grip. This suggests that cellulose is unlikely to diffuse spontaneously from the synthase. Currently, the glucan channel of BcsA is thought to bind weakly, if at all, to the growing strand, facilitating translocation (8). Only the acceptor-binding site at the transmembrane pore’s entrance has been suggested to contribute any grip and prevent backsliding (10). The observed strong binding and stall force may be a result of protein or product deformation disrupting the lubricating carbon-hydrogen π interactions and hydrogen bonding between the protein and cellulose (8). In a physiological setting, shearing forces or tension from increased cellulose interactions in the extracellular space can be greater than 13 pN and may stall biosynthesis, allowing for cleavage through hydrolysis and discouraging unproductive cellulose fabrication.

Fits to a Boltzmann energy barrier model (Fig. 4*A*) yielded a characteristic distance to the transition state associated with a mechanical transition of 4 nm, roughly corresponding to the length of BcsA’s transmembrane channel (∼4 nm), a length scale of approximately seven glucose molecules, or ∼27% of the length of the BcsAB complex (15 nm) (13). Another length scale of 5.0 nm was extracted from positional hopping seen in some motility traces under an apparent critical force of ∼2 to 3 pN (Fig. 4*D*). Such motion may originate from conformational changes in the machinery itself. A more likely possibility, however, is that hopping arises from changes to the product strand length during growth, such as a segment repeatedly folding back on itself and unfolding. While we use the Bell relationship to characterize the apparent two-state fluctuations (*SI Appendix*, Fig. S10), we note that, unlike defined transitions in nucleotide-based hairpins, cellulose folding inherently accommodates a distribution of states and structures and analysis of such should be interpreted locally. Additionally, abrupt extensions and retractions appear to be exponentially distributed in length (Fig. 4 *B*, *Top*), with fit parameters of 5.6 and 4 nm, respectively, suggesting a similar length scale is associated with a probability of forming such structures. In the presence of cellohexaose, the probability of forming structures increases, as well as the length scale of such structures.

The relative straightness of motility records was striking. Variance analysis is a method that relates the relative wandering of a record to constraints on kinetic models consistent with this motility profile. Here, one needs to assume a length scale associated with forward progress of a motility cycle. Assuming a length scale associated with the glucose repeat unit, 0.56 nm (34), variance analysis predicts the off-path (exiting the motility cycle and then returning) or multiple kinetic schemes for the biosynthesis cycle (43). However, if one assumes a length scale consistent with the Boltzmann fit and conformational change, ∼4 to 5 nm, a single rate-limiting step within the motility cycle is sufficient. Congruence between models is possible with a base unit of biosynthesis and single cellulose structure to be a seven-glucose segment, given that seven glucose bond additions per cycle result in a 4-nm increase of cellulose synthesis.

With a one-step kinetic cycle size of 4 nm, a distance to the transition state of 4 nm (Fig. 4*A*), an exponential fit length of 5.6 nm from the distribution of extensions (Fig. 4*B*), a hopping distance of 5.0 nm (Fig. 4*D*), and a persistence length of 6.2 nm (Fig. 5*D*), BcsAB appears to operate based on a unit length standard of 4 to 6 nm. The energetics of the polymerization cycle or the resulting cellulose structure upon extrusion may dictate this length scale.

One requirement of processive synthesis is transport. Other polymerases that have been measured at the single-molecule level, which include RNA polymerase (52), DNA polymerase (53), and the ribosome (54), utilize templates in their motility cycle and have the ability to interpret instructions for initiation, termination, etc. The temperature study revealed an activation energy of 32.5 k_B_T (80.5 kJ mol^−1^, *SI Appendix*, Fig. S1), of which glycosidic bond formation comprises 5 k_B_T (12.5 kJ mol^−1^) (36). Interestingly, the energy available from UDP-glc (17.4 k_B_T or 43.0 kJ mol^−1^) only supplies about half of the needed energy (55), which implies that two glucose additions may underly a cycle. Substrate-induced conformational changes at the enzyme’s catalytic pocket have been shown to be essential for polymer translocation (12). In addition, strand folding or cellulose-cellulose association likely helps drive transport of the newly synthesized strand, as evident by the modest increase in both bulk activity and single-molecule velocity of BcsAB in the presence of cellohexaose (Fig. 5 *G* and *H*). These interactions may also facilitate cellulose alignment and microfibril formation in plants and other bacterial species (56). Energetically, each hydrogen bond represents ∼1.6 k_B_T (4 kJ mol^−1^ or 6.6 pN·nm) (38). Conformational hopping observed in our experiments represent an exchange of work of ∼10 pN·nm, which represents approximately one to two hydrogen bonds. While energetically one must consider contributions from all bonds forming and breaking during a strand extension cycle, including contributions from solvation, the addition of two to four hydrogen bonds appears to be available to help drive transport of the nascent strand, especially through organization of cellulose microstructure. Because of this, bacterial cellulose synthesis may be encouraged in proximity to existing extracellular cellulose bundles.

Cellulose biosynthesis is dictated by the biochemical components available to BcsAB. Our studies directly test such biochemical elements’ impact on synthesis. Synthesis proceeds by addition of one glucose unit (12). Without the substrate, synthesis was halted as expected (Fig. 2*A*). Many glycosyltransferases are dependent on metal ion complexes to coordinate with UDP-glc and catalyze the reaction (12, 57). In our Mg^2+^ chelating experiments, motility slowed dramatically, yet some minimal synthesis appears to proceed (Fig. 2*C*). One explanation is that that coordination is not absolutely necessary, and some minimal synthesis is possible without Mg^2+^. However, it is also possible that our chelation experiments may have not completely removed Mg^2+^ from UDP-glc coordination.

It is well known that c-d-GMP is necessary for activating synthesis (8–10, 58, 59). BcsA recognizes an intercalated c-d-GMP dimer, with most of the interactions mediated by one nucleotide (10). Our studies suggest at least one c-d-GMP molecule maintains an incredible affinity toward BcsA. The population of the remaining fluorescent c-d-GMPf is consistent with a bond lifetime of 82.5 min and dissociation rate of 2.0 × 10^−4^ s^−1^ (Fig. 3*E*). Note that this is a lower bound due to potential photobleaching. Our studies were performed at dilute c-d-GMPf, 300 nM, where a shorter lifetime state might have been missed. In some cases, we did record traces where two molecules were bound to the same BcsA (*SI Appendix*, Fig. S3). Other groups have predicted much lower binding affinity, including c-d-GMP to BcsAB (association constant K_A_ = 1.8 μM) (9), to the bacterial ATPase MshE (K_A_ = 0.5 μM) (60), and to a mixture of bacterial c-d-GMP binding proteins (K_A_ = 7 μM) (61), but all were calculated from enzyme kinetics of bulk cellulose production, isothermal titration calorimetry, and pull-down assays, respectively. To calculate a K_A_ for comparison, we can assume the k_on_ is dictated by diffusion (estimated to be 10^6^ to 10^8^ M^−1^ s^−1^), yielding a K_A_ in the range of 2 to 200 pM (62). If we assume the binding rate is similar to that of ATP to kinesin (2 × 10^7^ M^−1^ s^−1^) or myosin (10^4^ M^−1^ s^−1^), we calculate K_A_s of 10 pM and 20 nM, respectively (63). However, ligand-receptor interactions that require conformational rearrangement have on rates closer to 10^2^ to 10^4^ M^−1^ s^−1^ (62), yielding a K_A_ in the range of 20 nM to 2 μM, well within the range of reported K_A_s. BcsAB undergoes large conformational changes with the aid of c-d-GMP, so it is within reason to suspect that a certain conformation is required for binding (10). Maintaining a specific K_A_ relative to other PilZ proteins is important for cell function (64). Additionally, temperature may also affect the binding kinetics of c-d-GMP to various proteins. In vivo, enzymatic degradation of c-d-GMP may contribute to release of c-d-GMP from BcsA and thus termination of cellulose biosynthesis (65).

Cellulose stretching experiments provide physical properties of the polysaccharide. The persistence length determined here was ∼6 nm (on the same length scale as the Boltzmann distance, transition distance, and extensions). This value is larger compared to other single-strand folding or clumping polymers, such as a polypeptide strand (0.4 to 0.6 nm) (66, 67), single-stranded RNA (0.91 nm) (68), single-stranded DNA (0.7 to 1.2 nm) (69), or polynorbornene (0.71 nm) (48). However, these values are much smaller than those seen in other ordered, thicker polymers, such as double-stranded DNA (47 nm) (45), double-stranded RNA (62 nm) (70), and amyloid fibers (1.5 μm) (46). Hydrogen bonding between adjacent glucose elements of the cellulose polymer is likely preventing swivel, resulting in a larger persistence length than similar polymers. Hysteresis was seen early on in stretching experiments with large opening distances (Fig. 5). Abrupt changes in position during motility traces suggest microstructures are present along the nascent polymer chain (Fig. 4*B*). We suspect single-strand cellulose is engaging in intrastrand hydrogen bonding and hydrophobic interactions, creating switchbacks and tangles, similar to microstructures observed in polynorbornene and tropocollagen (22, 48, 71). One may expect cellulose to readily form crystal-like structures if produced near other microfibrils, as seen in atomic force microscopy studies (72). The nonuniformity in size of extensions and retractions is attributed to the homogeneity in cellulose’s chemical composition. The extensions and retractions are not confined to a single length, location, or pairing registration. Cellulose retains some memory for how the chain was originally associated, as refolds are commonly the same size as the previous unfolding events. Eventually, repeat stretching physically tempers the strand, resulting in smooth, consistent physical properties (Fig. 5). At this point, cellulose maintains a very low axial stiffness (∼40 pN) compared to other biomolecules [ssRNA, 1,600 pN (68); ssDNA, ∼700 pN (69); and dsDNA, ∼1,100 pN (45)]. When cellohexaose binds, the resulting structure may include loops or other parallel structured regions that simply act as springs in parallel compared to the native chain. Assuming the whole contour contains parallel springs, this model suggests a limit to the equivalent spring constant K_eq_ = K_1_ + K_2_ of ∼80 pN (40 + 40 pN) for the hybrid strand. While this is greater than our measured stiffness of 68.5 pN, the measured strands likely contain segments where only one strand is present to sustain the load.

Cellulose produced by cellulose synthase complexes (CSCs) in plants (and some bacterial species) forms crystalline microfibrils containing multiple aligned cellulose polymers (73, 74). These microfibrils are unlikely to fold or exist in an entangled state. In contrast, our studies show that single-chain cellulose produced from individual BcsAB complexes appears compliant and amorphous and can actually fold on itself during strand synthesis. Previous transmission electron microscopy images of isolated cellulose synthases from *Gluconacetobacter hansenii* show no microfibril formation (31), despite successfully imaging microfibrils from isolated plant cellulose synthase rosettes (32), suggesting isolated Bcs synthases are unable to form microfibrils alone. Only high concentrations of surface-immobilized BcsAB synthases produce nonphysiological cellulose-2 fibers (72). Such elasticity and tendency to clump may play a crucial role in maintaining the biofilm’s gel-like structure and could also be critical for the coalescence of individual cellulose polymers into microfibrils. In biofilms of uropathogenic *E. coli*, amorphous and chemically modified cellulose acts as a mortar-like scaffold that maintains amyloid curli association and greatly increases bacterial adhesion strength to bladder cell surfaces (75). Higher-order cellulose production could jeopardize the biofilm’s structural, cohesive-adhesive, and protective qualities (18).

Our reported cellulose biosynthesis rates are slower than expected from bulk measurements in the literature, which includes a large range of reported rates (1.5 to 9 nm s^−1^) (31–33). While these measurements often do not determine the concentration of catalytically active enzyme for accurate rate measurements, our room-temperature conditions (21 °C) were lower than those of other investigations of cellulose production (25, 30, and 37 °C). The single-molecule experiments eliminate the availability of nearby cellulose microfibrils from assisting synthases, to which the extruded cellulose strand can hydrogen bond. The lack of available interstrand hydrogen bonds to assist with transport may also hinder synthesis rates. Additionally, other cellulose synthase subunits, such as BcsC and BcsD, are excluded from single-molecule experiments and are thought to play a crucial role in transport (8, 31). With the ability of cellulose to fold upon extrusion, shortening the apparent tether length during elongation, our apparent velocities represent the lower bound of possible biosynthesis rates. These, together with enzyme tethering, could explain the apparent slower synthesis rates observed (9, 32).

One key differentiation between BcsAB and cellulose synthases found in land plants is that the plant enzymes multimerize in a sixfold symmetry supramolecular CSC that is thought to produce an 18-strand microfibril immediately after synthesis (2, 58). Multimers likely exploit cellulose’s propensity to self-associate into microfibrils to form the load-bearing component of plant cell walls, while *E. coli*, for example, modifies cellulose polymers with lipid-derived phosphoethanolamine (76). The different cellulose biosynthetic machineries indicate evolutionary adaptations between the kingdoms to suit each one’s needs. Our studies provide the first insights into the physicochemical properties of individual cellulose polymers underlying a plethora of biological functions. The presented assay offers a foundation for future single-molecule studies on polysaccharide synthases, including trimer Bcs and plant CSCs, for which their products and functions are extraordinarily different (2, 73).

## Materials and Methods

### Materials.

See *SI Appendix*.

### BcsAB Expression and Purification.

The *R. sphaeroides* 2.4.1 BcsAB complex was expressed and purified as described (13). The purified complex was reconstituted into *E. coli* total lipid nanodiscs using the MSP1D1 scaffold protein, as recently described for related enzymes (77). In short, dried *E. coli* total lipid film was solubilized at a final concentration of 25 mM in buffer containing 20 mM Tris (pH 7.5), 100 mM NaCl, and 100 mM sodium cholate. The nanodisc reconstitution mixture was prepared according to a 1:4:160 molar ratio of BcsAB, MSP1D1 membrane scaffold protein, and lipid, respectively. Removal of detergents was initiated by the addition ∼200 mg mL^−1^ Bio-Beads SM2 (Bio-Rad), followed by incubation at 4 °C for 1 h. The same mass of Bio-Beads SM2 was added a second time, and the mixture was incubated at 4 °C overnight. The next day, the same mass of Bio-Beads SM2 was added, followed by incubation at 4 °C for 1 h. After removal of Bio-Beads SM2, the nanodisc-reconstituted BcsAB complex was incubated at room temperature for 15 min with 5 mM UDP-glc, 20 mM MgCl_2_, and 30 μM c-d-GMP to synthesize cellulose tether. After that, the complex was purified on a Superdex 200 column equilibrated in nanodisc gel filtration buffer containing 20 mM Tris (pH 7.5) and 100 mM NaCl. Peak fractions containing the BcsAB complex were snap frozen in liquid nitrogen until further use.

### Motility and Stretching Assay Preparation.

We use one primary assay construction for both synthesis and stretching measurements. Flow cells are made with double-sided tape between a glass slide and a KOH-etched glass coverslip. A single BcsAB complex bound in a His-tagged nanodisc is fixed to the glass coverslip using nonspecifically bound streptavidin (0.1 mg mL^−1^) and a biotinylated anti-His antibody construct (0.01 mg mL^−1^). A 5 mg mL^−1^ solution of casein is incubated to block nonspecific binding after streptavidin placement and before BcsAB attachment. To prevent mixing of reagents, a solution containing 5 mg mL^−1^ bovine serum albumin and 1 mg mL^−1^ casein is washed between incubation steps. Surface-bound BcsAB complexes (600 pM) are incubated with 1.09-μm polystyrene beads completely coated with cellulose-binding DNA aptamers via biotin/streptavidin interactions, allowing for the beads to bind to the extruded cellulose chain. Afterward, a complete synthesis buffer (pH 7.5) containing 25 mM NaH_2_PO_4_, 50 mM NaCl, 10 mM cellobiose, 10% glycerol, 5 mM UDP-glc, 30 μM c-d-GMP, and 20 mM MgCl_2_ is flowed into the flow cell directly before data acquisition. Control buffers comprise the same ingredients as the complete synthesis buffer, except excluding one component in each case. Mg^2+^ control buffer includes 50 mM EDTA. All experiments were conducted at 21 °C. During control experiments, the flow cell is left open, and midexperiment, 45 μL of control buffer exchanges with the complete synthesis buffer before being washed out again. For hybridization experiments, cellotetraose (5 and 50 mM) and cellohexaose (0.45 mM) were included separately in the motility buffer, maintaining the final concentrations of the complete synthesis buffer. The concentrations of cellotetraose and cellohexaose were determined by the available supply and the solubility limit, respectively.

### Motility Data Acquisition and Analysis.

Once the flow cell is loaded on the microscope, beads are trapped by a 1,064-nm laser and calibrated for trap stiffness and position within our detector zone. Tethered beads are centered over the complex and subsequently displaced by moving the piezo stage. In the instances where the tether shows evidence of folding and microstructure formation, we continue to move the stage as the tether undergoes extensions and mechanical relaxation before we record biosynthesis rates. As cellulose is synthesized, bead position is recorded at 5 kHz for as long as 10 min. Fiducial 0.75-μm polystyrene beads bound to the coverslip facilitate drift tracking and correction through a custom cross-correlation video tracking algorithm similar to Brady et al. (36). Motility traces are corrected for drift and then decimated to 100 Hz. The actual tether length is calculated with a correction factor of 1/sin(θ), where θ is the incidence angle (angle from a line perpendicular to the coverslip surface) (50). Custom MATLAB scripts determine a velocity and average force for the entire trace. Velocities of less than 0.01 nm s^−1^ were considered an absence of synthesis. For the force-velocity curve, velocities were binned by force and averaged every 2 pN. Each bin was weighted corresponding to the number of items in each bin for the fit. We fit the weighted, averaged velocities to the general Boltzmann relationship (28). Abrupt extensions or retractions were located by a sliding step-finding MATLAB script that distinguished changes in mean position greater than two SDs from the previous segment’s mean (78). The two sliding segments were 0.2 s in size. Typical force ranges for all control experiments were 3 to 8 pN, within the active range unaffected by force. The randomness parameter was calculated individually for each trace from the variance from the mean trajectory and averaged to find the mean randomness parameter for each given characteristic distance.

### Dye-Labeled c-d-GMP Bulk Activity Control.

To assess the activity of the purified, nanodisc-reconstituted BcsAB complex in the presence of DY-547–labeled c-d-GMP (c-d-GMPf), 0.01 mg mL^−1^ protein was incubated overnight at 37 °C in the presence of 5 mM UDP-glc, 20 mM MgCl_2_, 0.25 µCi ^3^H-labeled UDP-glc, and 30 μM c-d-GMP, 30 μM c-d-GMPf, or no c-d-GMP. After synthesis, the reaction mixture was subjected to paper chromatography and liquid scintillation measurements to quantify the amount of product, as previously described (9). Each reaction was carried out in triplicate (*SI Appendix*, Fig. S2). To test the bulk activity of BcsAB in the presence of cellohexaose, a similar protocol was followed. The protein was incubated with all reaction components in the presence or absence of 0.45 mM cellohexaose overnight.

### Stretching Data Acquisition and Analysis.

During cellulose stretching experiments, tension is increased by moving the sample at a velocity of 64 nm s^−1^ and at loading rates between 2.25 and 8.5 pN s^−1^, depending on trap stiffness, until the bead is pulled out of the trap center. The stage direction is then reversed to relax the polymer until the bead is centered over the complex again. To avoid tether beads from sticking to the coverslip, the bead is slightly lifted off the surface. The tether length is calculated using the same correction factor as in motility experiments (50). Trap stiffness varied with laser intensity. The position of the bead relative to the trap is sampled at 5,000 Hz and averaged every 4 nm. The extension of the polymer is calculated from stage position measurements, angle correction for assay geometry, and bead position data. We can assume the mechanical properties of BcsAB are negligible because the size of the complex (∼15 nm) is much less than that of the strand (∼1 μm). Multiple sequential stretches are acquired in a single run to observe hysteresis and mechanical relaxation and to monitor the relative changes in apparent contour length over time. The elevation of the bead from the surface does not affect the change in apparent contour length. A custom MATLAB script was used to fit stretching curves to the eWLC model (51), taking into account the assay geometry angle. Unfolding distances of cellulose were determined by calculating the difference in contour lengths before and after an elongation event.

DNA tether controls were performed by nonspecifically binding streptavidin to the glass coverslip and incubating with casein, as done in BcsAB assays. Next, a solution of 30.7 ng mL^−1^ biotin-3,500 bp DNA-digoxygenin was incubated, followed by antidigoxygenin-coated beads to form coverslip-tethered beads. The DNA constructs and beads were made using a protocol outlined in Banik et al. (79). The rest of the experiment and analysis mimicked that of cellulose experiments exactly.

### TIRF Measurements.

Synthase surface attachment methods were the same as the motility assay preparation described above. However, a 1% biotin-polyethylene glycol (PEG) coverslip replaced the KOH-etched coverslip to prevent nonspecific binding of fluorophores, and polystyrene beads were excluded. Assay design and signal measurements are similar to those reported by Shin et al. (80). Synthases were incubated for 20 min in the complete synthesis buffer containing 300 nM DY-547–labeled c-d-GMP (Biolog) and 29.7 μM unlabeled c-d-GMP. The synthesis buffer also included an oxygen-scavenging mixture of 0.8% glucose, 165 U/mL glucose oxidase, and 2,170 U/mL catalase in Trolox to minimize photobleaching.

Immediately before data acquisition, c-d-GMPf was washed out with 10 times the flow cell volume (10 × 20 μL) of normal synthesis buffer. The sample was illuminated by a 532-nm laser only during image sampling. A laser power of 40 μW illuminated a field of 3,000 μm^2^. Images of the specimen plane were collected at 0.33 Hz for 1 h with Andor’s iXon camera via a triggering mechanism to illuminate the sample for 100 ms only during each frame of image acquisition, culminating in 120 s of total exposure. Custom MATLAB scripts identified and measured the lifetime of bound fluorescent c-d-GMP. Spots with brightness that varied or drifted considerably were excluded. Single-molecule fluorescence was identified by a steep photobleaching or unbinding event in which the signal returned to baseline. For those that did not photobleach or unbind, only spots with a brightness signal consistent with the single-molecule events were considered.

## Supplementary Material

Supplementary File

## Data Availability

All study data are included in the article and/or *SI Appendix*.
