# Supplementary material for: Single-molecule investigations of single-chain cellulose biosynthesis
Source: Proc Natl Acad Sci U S A. 2022 Sep 26;119(40):e2122770119. doi: 10.1073/pnas.2122770119 (PMC9546554; doi:10.1073/pnas.2122770119)
Supplement: Supplementary File [file pnas.2122770119.sapp.pdf]

PNAS
www.pnas.org

Supplementary Information for
Single-molecule investigations of single-chain cellulose biosynthesis

Mark A. Hilton, Harris W. Manning, Ireneusz Gorniak, Sonia K. Brady, Madeline M. Johnson, Jochen Zimmer,* and Matthew J. Lang*

*Correspondence, Jochen Zimmer and Matthew J. Lang
Email: jz3x@virginia.edu, matt.lang@vanderbil.edu

This PDF file includes:

Supplementary text
Figures S1 to S17
Tables S1 and S2
SI References

Supplementary Information Text

Materials. Bovine Serum Albumin (EMD Millipore – 12657), Casein (Sigma – C7078), biotinylated anti-his antibody (Qiagen - 34440), streptavidin (EMD Millipore – 189730), cyclic-di-GMP (BioLog – C057-01), UDP-glucose (Sigma – U4625), ^{3}H-labelled UDP-glc (PerkinElmer NET1163250UC), $MgCl_2$ (Mallinckrodt – 5958), cellobiose (Acros Organics – 108460250), glycerol (Sigma – G7893), 1.09 mm streptavidin-coated polystyrene beads (Spherotech – SVP-10-5), 0.75 mm polystyrene beads (Spherotech – PP-08-10), biotinylated cellulose-binding DNA aptamer (IDT 5'-biotin/GCG GGG TTG GGC GGG TGG GTT CGC TTG GCA GGG GGC GAG TG-3'), PBS (1x pH 7.4), Tris-HCl buffer (RPI – 1185-53-1, 20 mM pH 7.0), purified bacterial cellulose synthase BcsAB complexes reconstituted into nanodiscs, NaH_2PO_4 (Mallinckrodt – 7892), NaCl (Sigma – S7653), deionized water, EDTA (Sigma E6758), biotin-3500 bp DNA-digoxygenin (1), protein G-coated 1.09 mm polystyrene beads (Spherotech – PGP-08-5), anti-digoxygenin, KH_2PO_4 (Mallinckrodt – 7100), Na_2HPO_4 (Mallinckrodt – 7917), DY 547-cyclic-di-GMP (BioLog – D116), mPEG-silane MW 5,000 (Laysan Bio), biotin-PEG silane MW 5,000 (Laysan Bio), KOH (Fisher Chemical), β-D-glucose (Calbiochem – 34635), glucose oxidase from *Aspergillus niger* (Sigma – G2133), catalase from bovine liver (Sigma – C100), Trolox (6-hydroxy-2,5,7,8-tetramethylchroman-2-carboxylic acid) (Sigma – 238813), cellohexaose (Megazyme – O-CHE), cellotetraose (Megazyme – O-CTE-50MG).

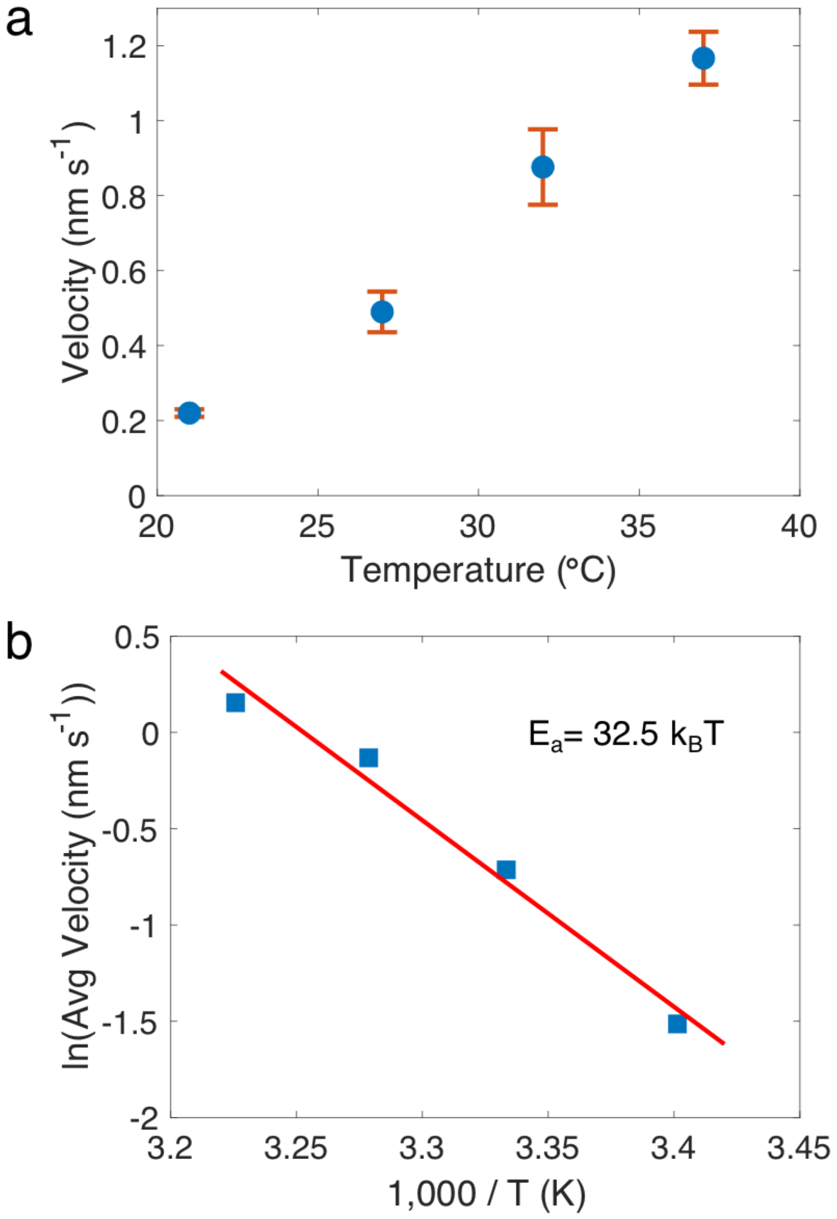

Figure S1. Cellulose biosynthesis at varying temperatures. (a) There is a significant increase in activity with increasing temperatures. The velocity of synthesis at 27°C is 0.49 ± 0.05 nm s^{-1} (SEM, N=25), at 32°C is 0.87 ± 0.10 nm s^{-1} (SEM, N=20) and at 37°C is 1.2 ± 0.1 nm s^{-1} (SEM, N=50). Error bars are SEM. (b) An Arrhenius fit of BcsAB motility from 21°C to 37°C yields an activation energy of 32.5 k_BT (80.5 kJ mol^{-1}).

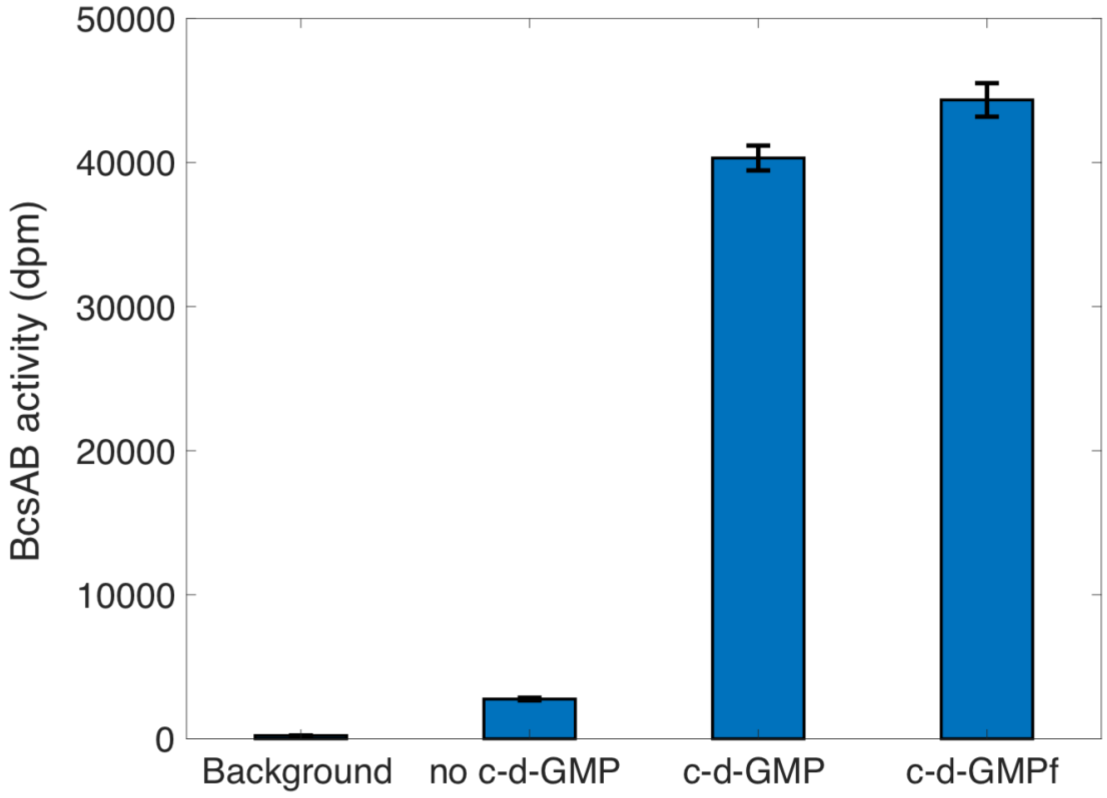

Fig. S2. C-d-GMP vs c-d-GMPf bulk synthesis rates. UDP-glc uptake rates in disintegration per minute of BcsAB (dpm). The background contained no BcsAB. The control without c-d-GMP shows a significant reduction in activity, while there is no significant difference between synthesis rates in the presence of c-d-GMP versus c-d-GMPf. Error bars denote SEM.

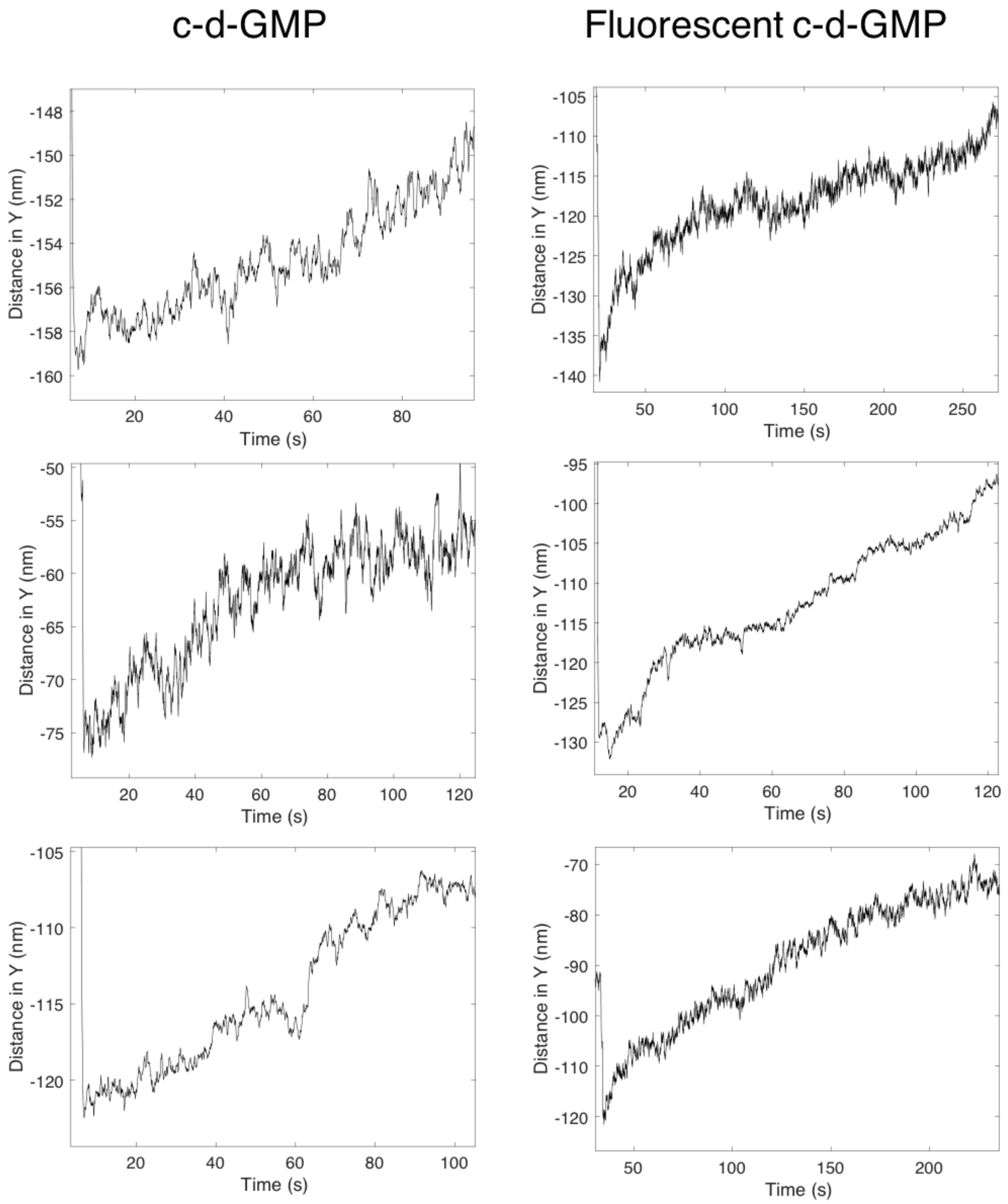

Fig. S3. C-d-GMP vs c-d-GMPf single-molecule synthesis rates. Example traces of single-molecule cellulose synthesis under normal synthesis buffer conditions (left column) and with c-d-GMPf replacing c-d-GMP (right column). For this experiment, all measurements were between 4-8 pN of force. The mean velocity was 0.25 ± 0.02 nm s^{-1} (N=39, SEM) under normal conditions and was 0.25 ± 0.3 nm s^{-1} (N=8, SEM) in the presence of c-d-GMPf. The fluorescent dye had no apparent effect on single-molecule cellulose synthesis.

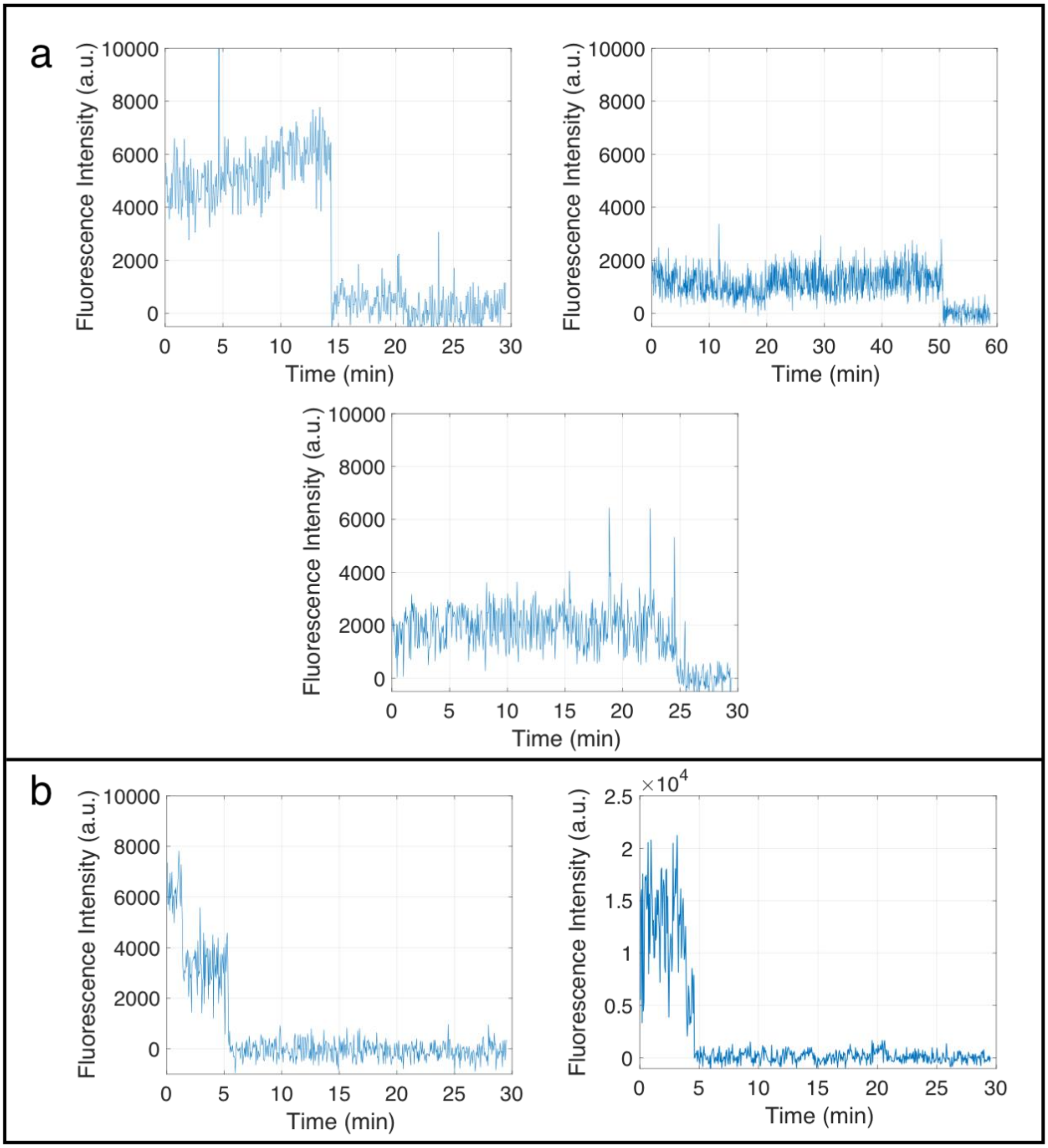

Fig. S4. Example fluorescence traces. (a) Example fluorescence traces show high signal to noise ratio and single photobleaching/dissociation steps indicating single-molecule events. Only signals that remained constant at a brightness consistent with single-molecule fluorescence were considered. Note in these experiments the intensity of the excitation laser is not uniform over the whole field of view, resulting in a range of spot brightness levels. Single-molecule fluorescence brightness criteria were appraised from the brightness of single photobleaching events as well as from previous experiments (2). (b) Few traces contained two photobleaching/dissociation steps representative of two c-d-GMPf binding to the activation site.

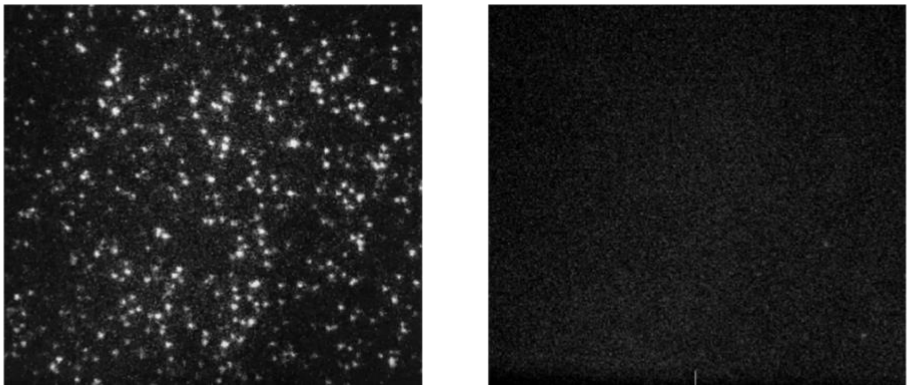

BcsAB	+	-
1 nM DY547 c-di-GMP	+	+
30 µM Unlabeled c-di-GMP	+	+

Fig. S5. TIRF control. To test the non-specific blocking capabilities of our PEG coverslip and ensure no fluorescently labelled c-d-GMP in solution triggered a response, we ran our assay with and without BcsAB, to which c-d-GMP binds. With BcsAB, we see many spots indicating c-d-GMPf is binding near the surface. Spots vary in intensity as two c-d-GMP bind to the PilZ domain and as the laser intensity varies across the specimen plane. Without BcsAB, we see no signal nor non-specifically bound c-di-GMPf near the surface. We can conclude that a fluorescent signal originates from c-d-GMPf bound to BcsA.

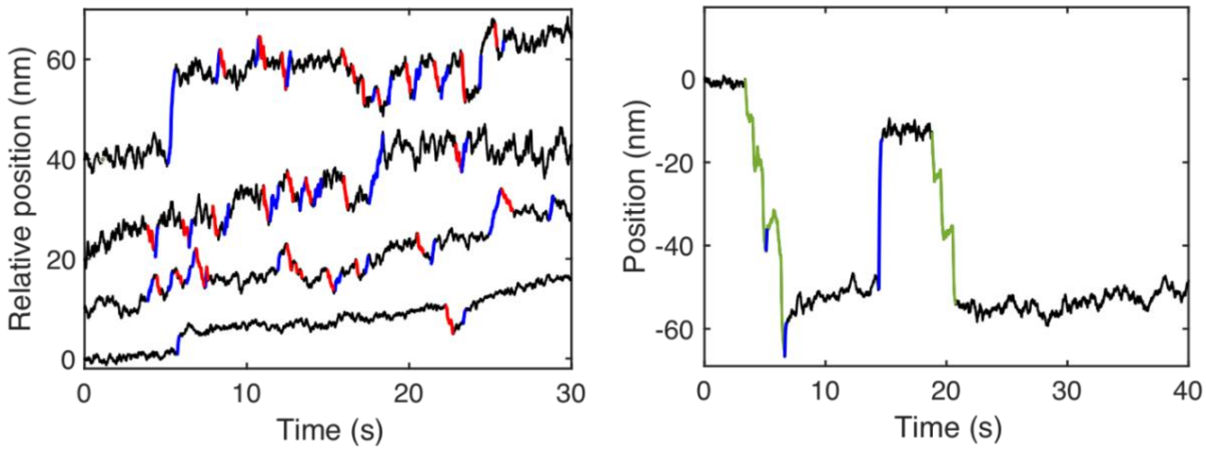

Fig. S6. Extensions and retractions. During motility traces, abrupt extensions (blue) and retractions (red) are seen. Some examples are shown. Extensions and retractions vary in size (2-100 nm) with smaller events shown on the left (2-16 nm) and larger on the right (10 and 40 nm). Position changes due to stage translation are in green.

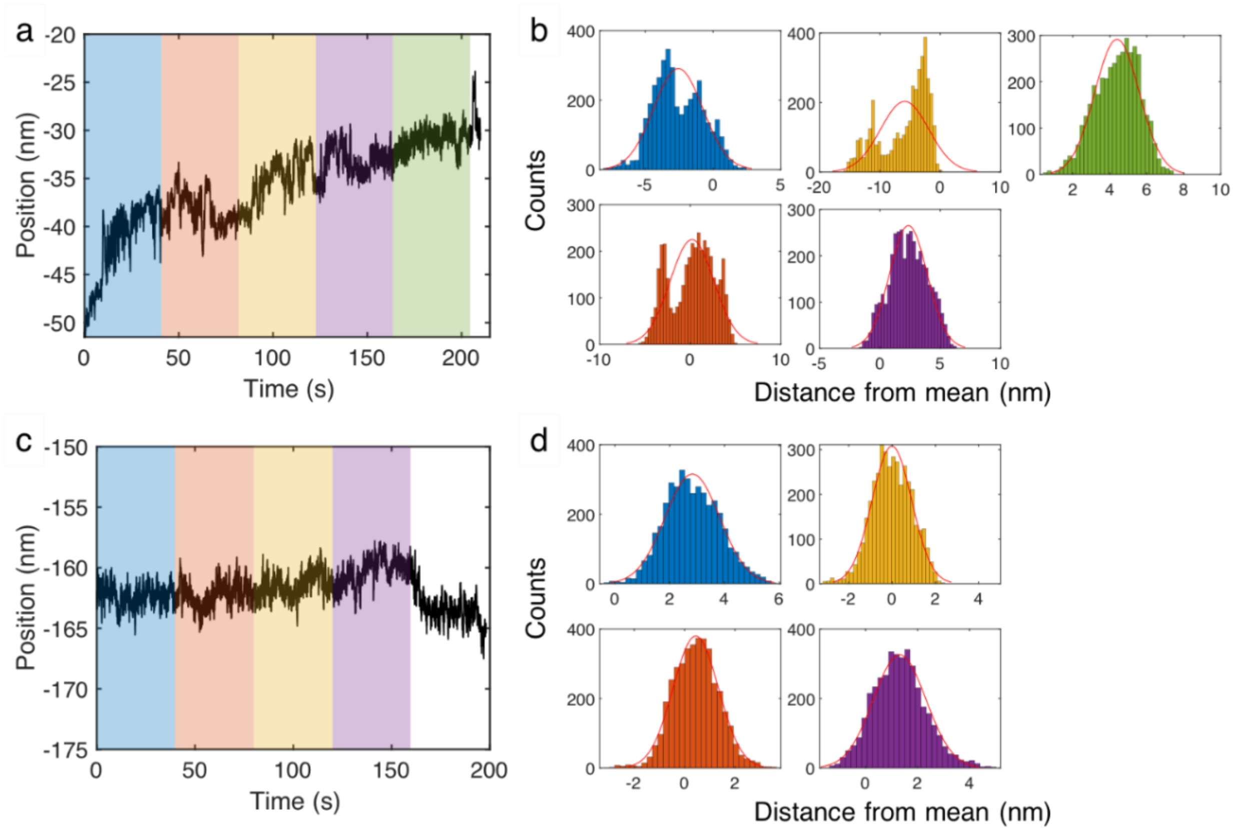

Fig. S7. Cellulose vs DNA position distributions. (a) An example trace of cellulose synthesis under approximately 2 pN of tension segmented into 40-second intervals for analysis. (b) The position distributions from the mean over each time interval. Some segments display gaussian behavior, while 75% show evidence of repeated jumps (N=25). Distributions are color coded to match the respective segment on the trace. A gaussian fit is shown for comparison purposes. (c) An example trace of a DNA tether under approximately 5 pN of tension also segmented into 40-second intervals. (d) The corresponding position distributions for each segment. All distributions for DNA traces (N=16) show gaussian behavior. Segments maintained a predetermined size for accurate comparisons, which excludes the remainder of each trace when not divisible by the time window.

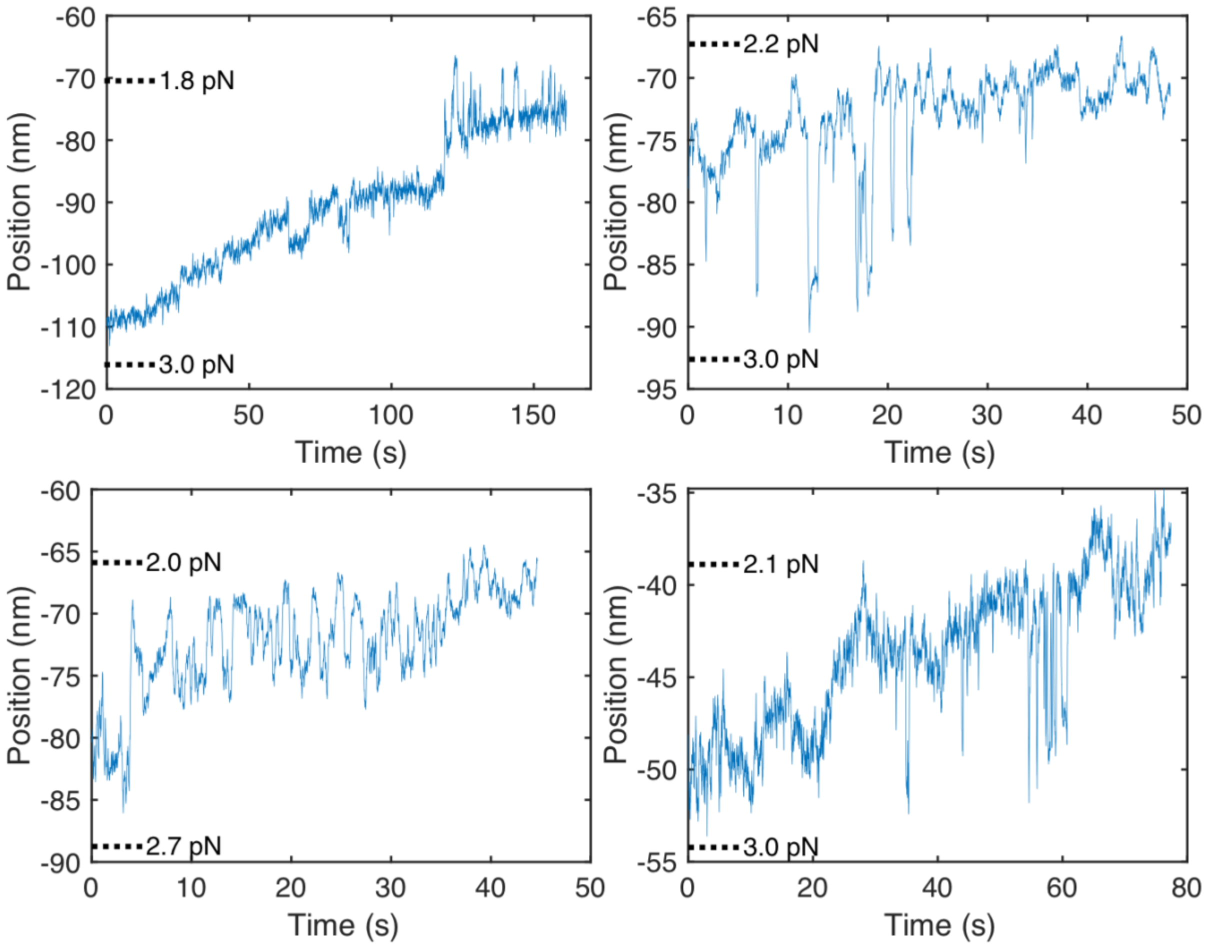

Fig. S8. Example traces with rapid reversible transitions. Above are example traces in which reversible and rapid structural transitions are apparent. We observed a total of 51 out of 201 traces that exhibit this transition behavior. The transitions range from 3-10 nm in size and are typically only seen at a force range of 1-4 pN. The mean frequency of elongation transitions is 4.0 s^{-1}, while the mean retraction frequency is 4.1 s^{-1}. We note reference forces for the given distance from the center of the trap to show the applied force conditions during synthesis.

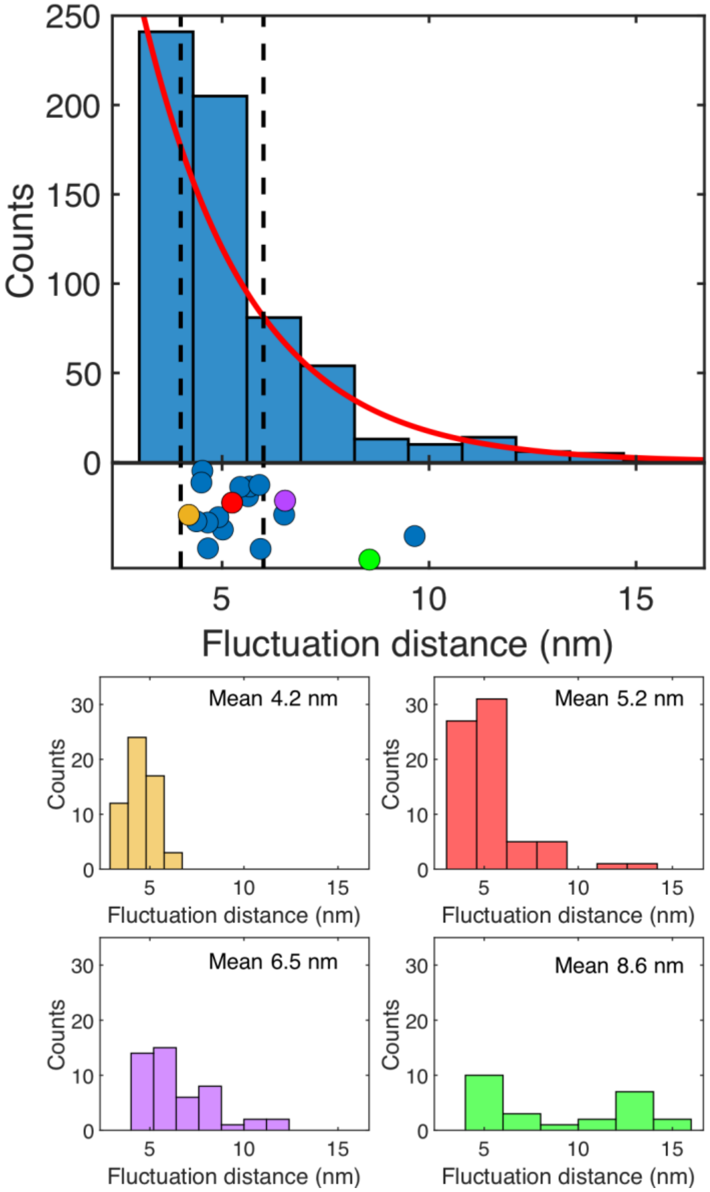

Fig. S9. Histogram of observed fluctuation distances. Distances between gaussian peaks from bimodal, 5-second segments represented by Fig. 4d. We see a range of 2.3 nm to 15 nm in repeated jump sizes with >95% occurring between 2.3 nm and 9 nm. We assume that there are upper and lower limits to the size of folds while under tension. The lower limit, determined by hydrogen bonding along the polymer chain, steric clashes and bending restrictions prevents small folds, while the upper limit is defined by the work required to close a fold under tension, the probability of which will grow exponentially with the size of the fold. *In vivo*, larger folds are certainly possible, as seen from initial pulls on cellulose tethers grown without tension (*SI Appendix* Fig. S6). The scatter plot below shows the mean fluctuation distance of each molecule represented by a single point. Representative distributions for a few points are shown in their corresponding color. A majority (14 of 18) of the molecules have mean fluctuations between 4 and 6 nm, with the range denoted by black dotted lines. The yellow and red molecules are considered for the Bell analysis while still limiting all events to 4-6 nm. The purple and green molecules were excluded.

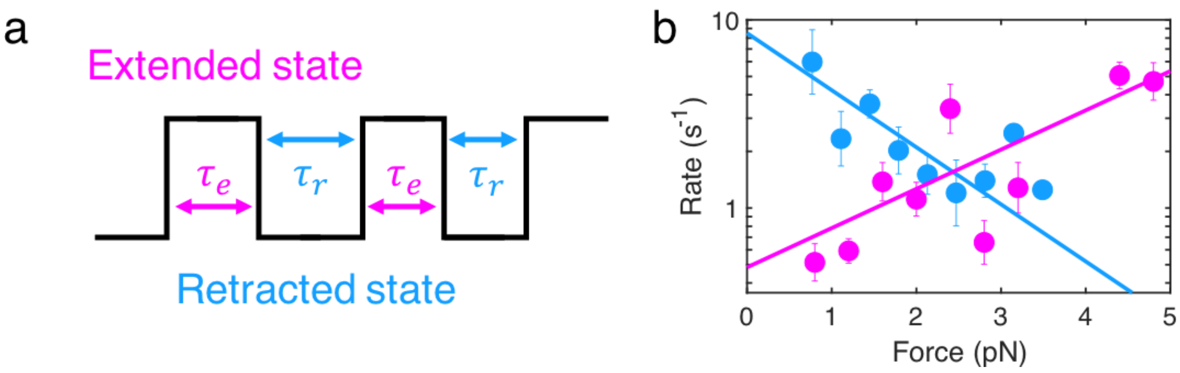

Fig. S10. Transition frequency as a function of force. (a) Schematic of a reversible hopping between extended and retracted states. (b) Extension/retraction rates vs force for all non-gaussian, bimodal segments (N=322 events over 14 traces) fit to a logarithmic equation of the Bell model: $k=k_1\exp(\Delta xF/k_bT)$. Only segments with a hopping distance within the range of 4-6 nm were considered, as the rates will change with transition distance. The intersection of fits reveals an equilibrium force of 2.4 pN, but both transitions share similar rates over the 2-3 pN range.

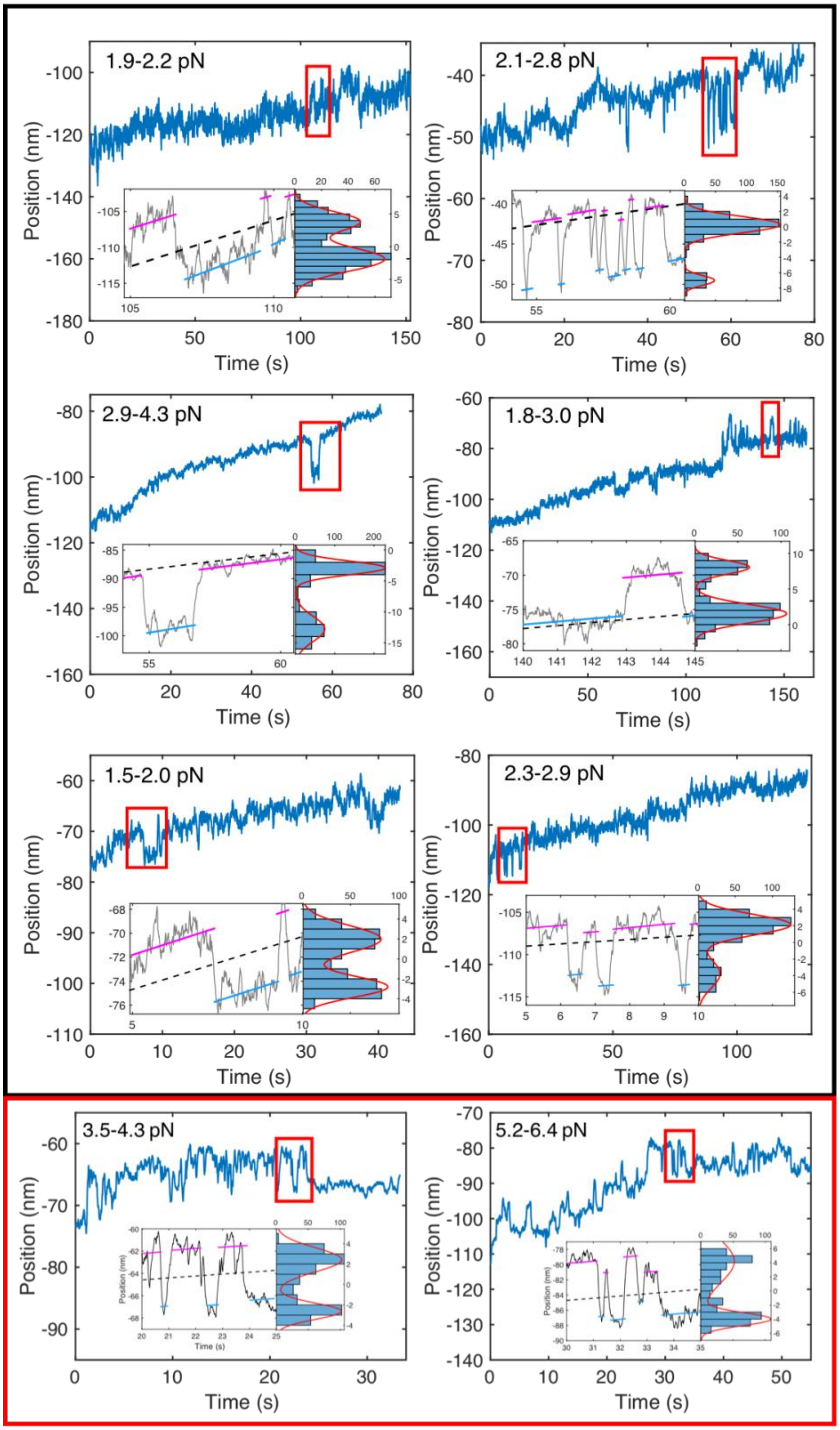

Fig. S11. Reversible transition analysis examples. Example traces with segments containing reversible transitions that were used to measure the lifetimes in both extended and retracted states. The insets show the red-boxed portions of the full trace. The black dotted line denotes the mean trajectory while the

histogram shows the distance from the mean trajectory, similar to Fig. 4d. The histograms show bimodal behavior between an extended state and retracted state. The extended (pink) and retracted (blue) states are outlined in the inset. The lifetimes of each state were measured to find a transition rate between states. A total of 14 traces and 322 events with fluctuation distances between 4-6 nm were considered. We note force ranges for each trace. As cellulose is synthesized, the bead is drawn towards the trap center and the force applied weakens slightly during the trace. The black box around the first six examples denotes no cellohexaose, and the red box around the last two examples indicates the presence of cellohexaose. Rapid transitions also occurred in the presence of cellohexose, here in the 4-6 pN range. The mean transition size for these particular events was 5.9 nm and the mean rate of transition was 0.8 s^{-1} (N=110 events over 2 molecules).

a $$r_{trajectory} = \lim_{t \to \infty} \frac{< (x(t) - < x(t) >)^2 >}{d < x(t) >} = \frac{slope\ of\ variance}{d \times slope\ of\ trajectory}$$

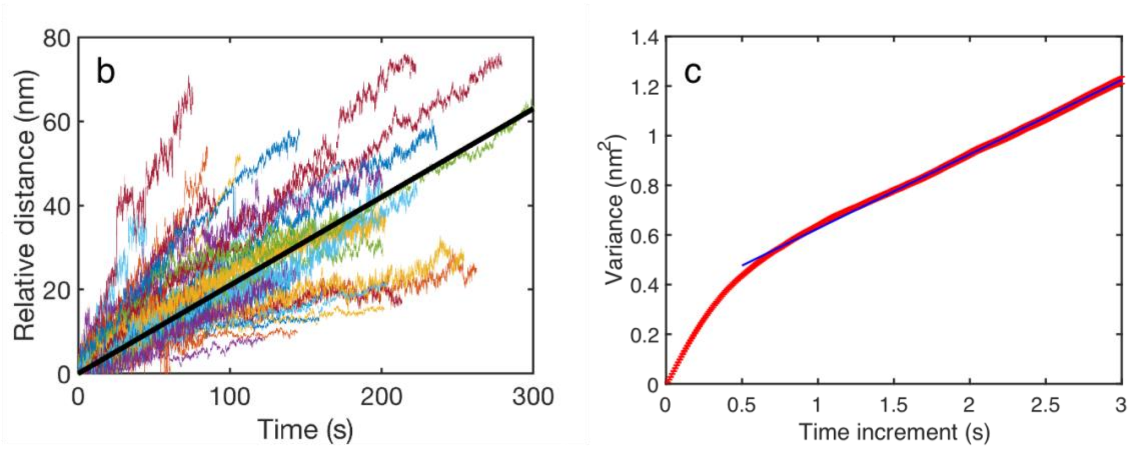

d

Step Size (*d*) (nm)	Randomness Parameter (± SEM)	Number of rate limiting steps
0.56	6.75 ± 0.84	NA
4	0.94 ± 0.12	1

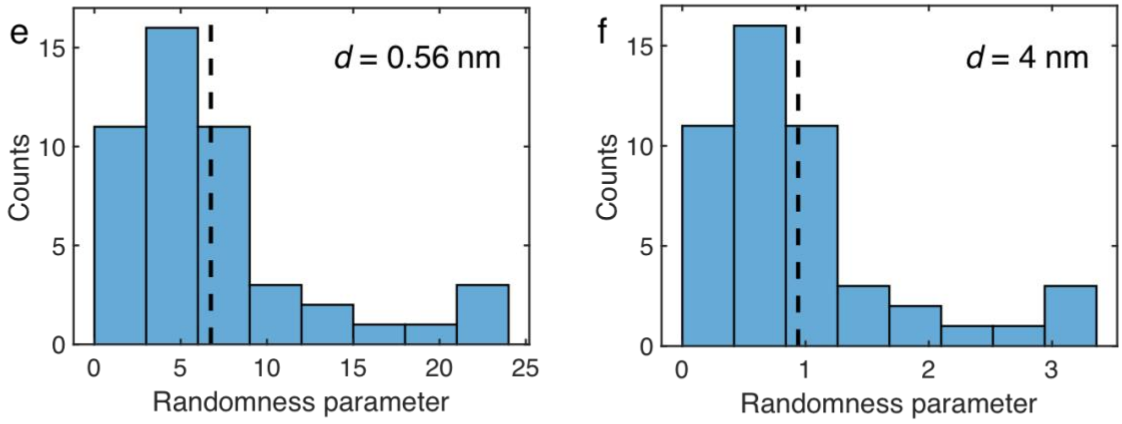

Fig. S12. Variance analysis. From a collection of motility traces, we calculated the variance from each mean trajectory, and, using the equation in (a), revealed a randomness parameter for each motor sampled (N=50). (b) A selection of motility traces in various colors in which each trace's slope of trajectory was used to find its randomness parameter. The black line indicates mean velocity. (c) An example variance calculation of a motility trace over increasing time increments. The slope of variance is used in equation (a) as well as both the monomer size and the distance between transition states from the force-velocity fit as the step size *d* in equation (a). (d) A step size of one glucose unit (0.56 nm) yields a randomness parameter of 6.75 ± 0.84 (SEM) suggesting a complex off pathway kinetic may underpin motility, while the distance between transition states (4 nm) indicates a randomness parameter of 0.94 ± 0.12 (SEM) representative of one rate limiting step. Histograms of the randomness parameters for step sizes of 0.56 nm (e) and 4 nm (f) show the spread in the variance parameter exhibited by the collection of molecules compared to the mean randomness depicted by a dashed line. Multiple kinetic schemes create higher likelihood of dynamic disorder likely giving rise to the spread between molecules (3).

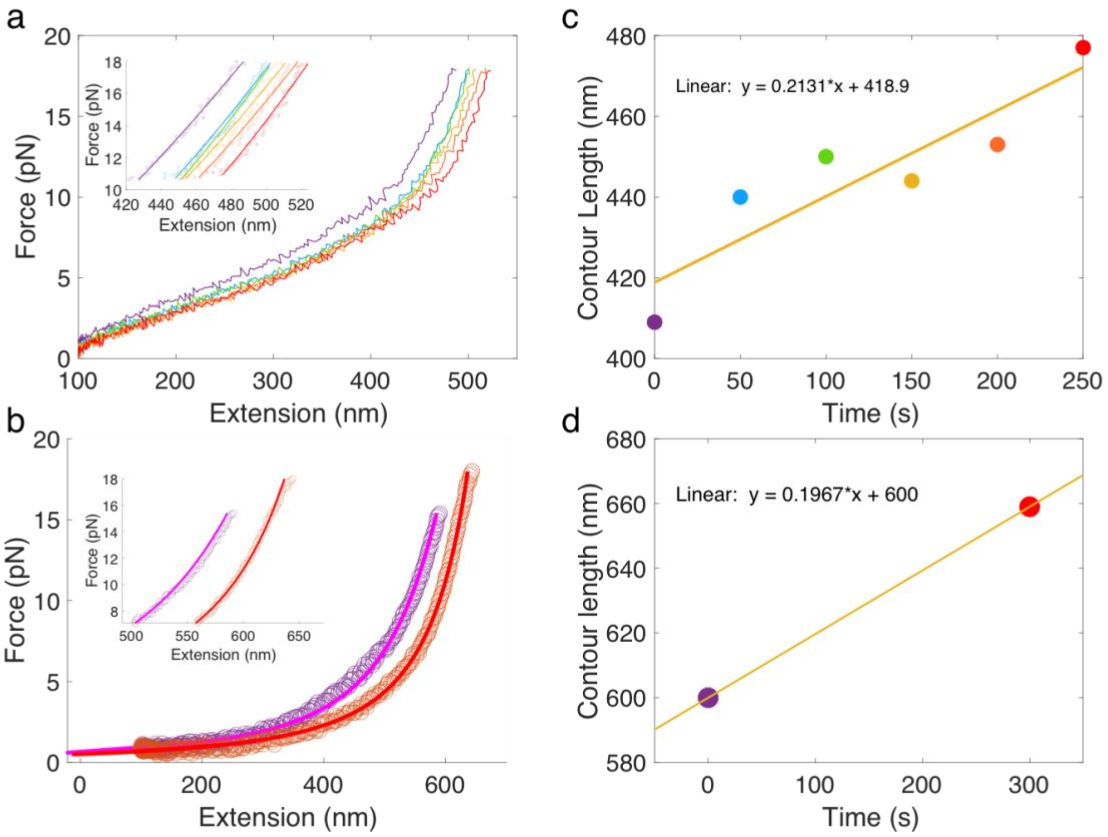

Fig. S13. Unloaded velocity. We measured the velocity of synthesis using a second method in which we measured the contour length of a growing strand of cellulose over time. As time progresses, the color scheme changes from purple to red. The consecutive stretches seen in (a,b) were fit to the eWLC model, and contour lengths were plot against time (c,d). to extrapolate a velocity. We performed this experiment 6 times revealing an average unloaded velocity of 0.32 ± 0.07 nm s^{-1} (SEM), similar to that observed from motility data. For these experiments, measurements are typically performed slightly off the glass surface to prevent nonspecific sticking. This slight pulling up in the z direction will impact the slope of the entropic region of the curve as seen in (a) for the region spanning 100-400nm in contrast to the flatter region seen in (c). The relative flatness of the entropic region does not impact the ability to determine a change in apparent contour length which depends on the steeper rise location towards the end of each stretching curve.

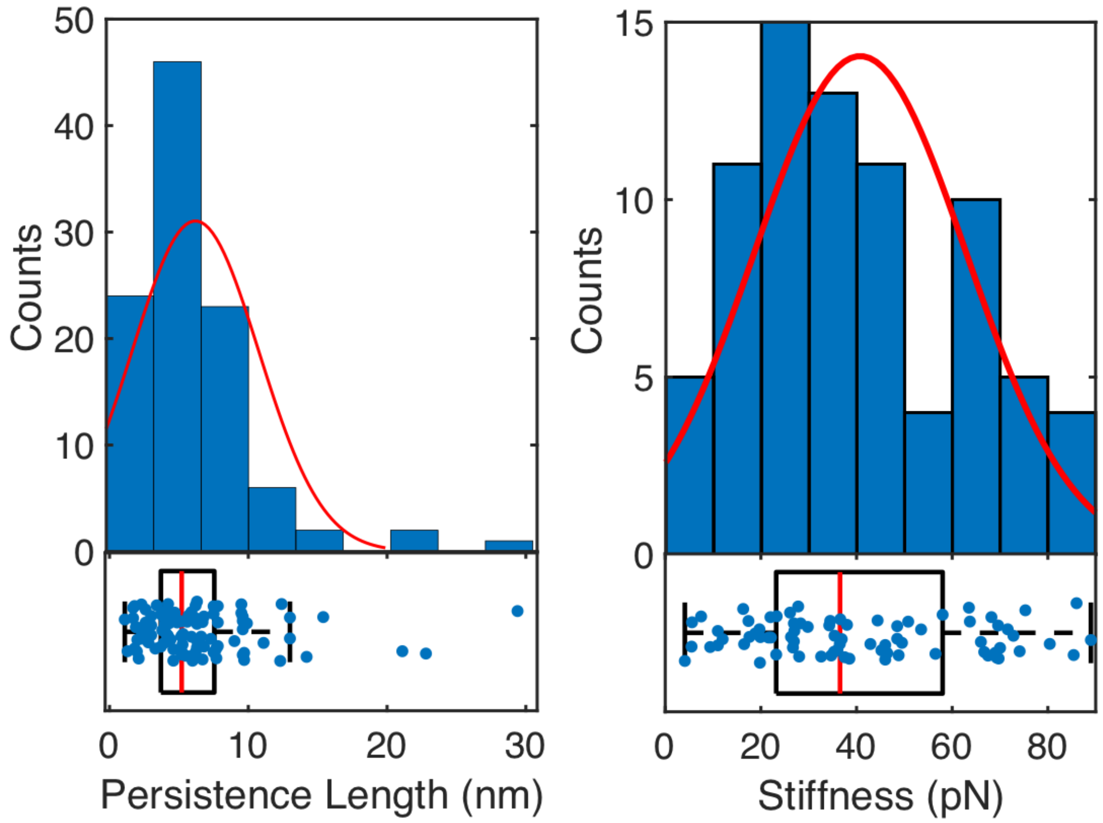

Fig. S14. Persistence length and axial stiffness. Above are histograms of the persistence length and stiffness measured from cellulose stretching experiments, and below are boxplots showing distributions. Observed persistence lengths converge to gaussian behavior and reveal a mean persistence length of 6.2 ± 0.4 nm (SEM, N=104). Stiffnesses have a much wider distribution, but still exhibit gaussian behavior. The average stiffness was 40.7 ± 2.5 pN (SEM N=78). The contour length of the strand had no effect on the measured stiffness. The range is possibly due to irregularities in cellulose microstructure.

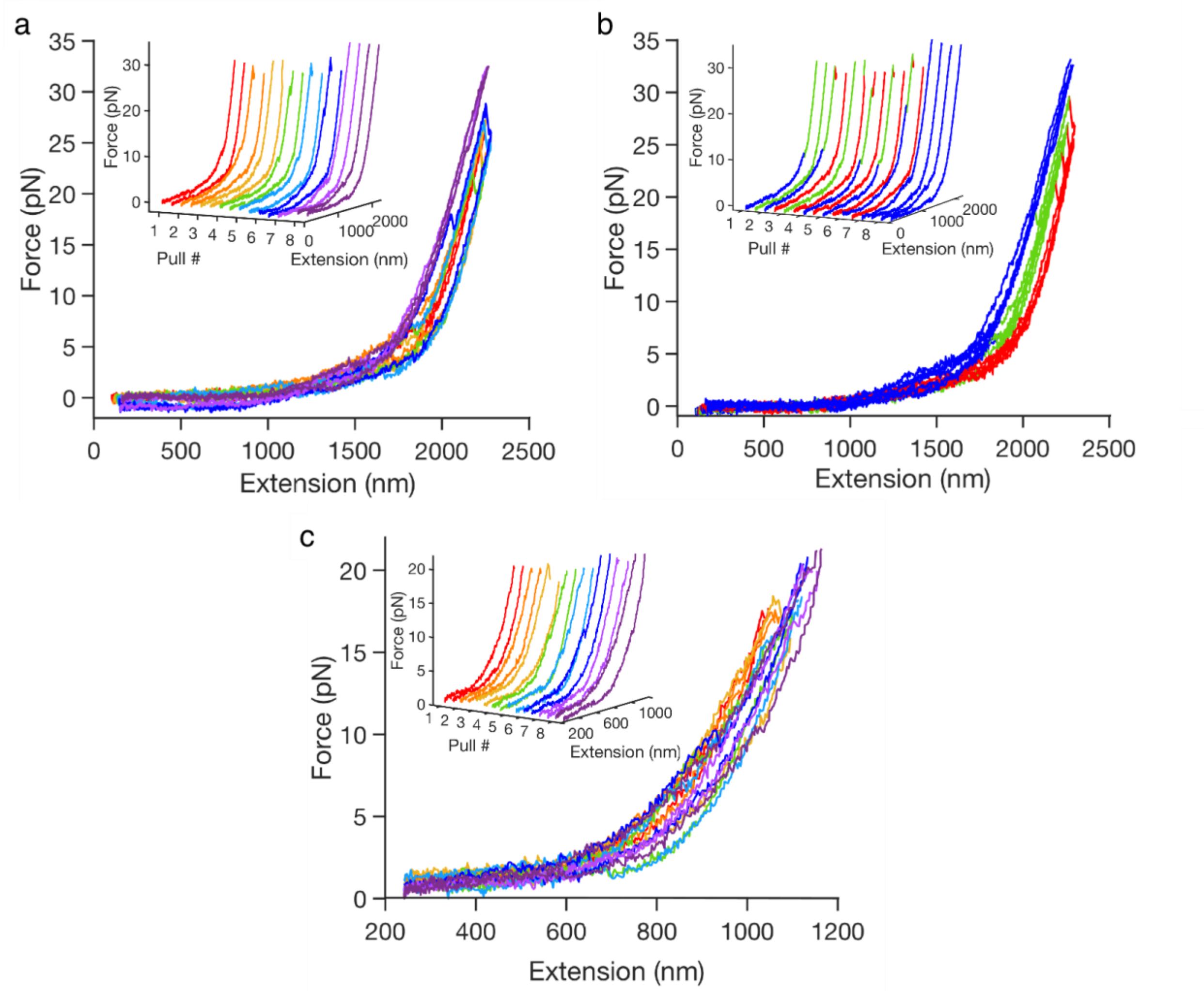

Fig. S15. Cellulose stretching hysteresis with cellohexaose. Subsequent stretching of cellulose in the presence of 0.45 mM cellohexaose revealed multiple refolding events, preventing the generation of a stable tempered state, even under force. All figures show a side view overlapping sequential cellulose-cellohexaose hybrid stretching curves, while the insets display a diagonal view to better track the relative pull sequence of each individual curve. (a) Repeated cellulose stretching curves are shown to progress in time from red to purple. In this example, the final four curves displayed stable states with shorter contour lengths, indicating cellulose-cellohexaose hybrid microstructure is secure even under 30 pN of force. This behavior is absent in isolated single cellulose strands (Fig 5a). (b) State-based rendering of the same cellulose-cellohexaose hybrid as in (a) appears to follow three local minima, evident by the blue, green, and red force-extension pathways. Designation of specific states is unique to the specific hybrid strand and varies between strands. The hybrid strand in (c) shows a great number of possible states and frequent transitions between them. Each hybrid strand exhibited the ability to adopt smaller apparent contour lengths and more compact configurations under considerable force, further highlighting the predisposition for self-binding within the strand. Additionally, the probability at which microstructure reforms between stretches appears much greater with cellohexaose present than without, indicating a larger than expected propensity to form microstructure.

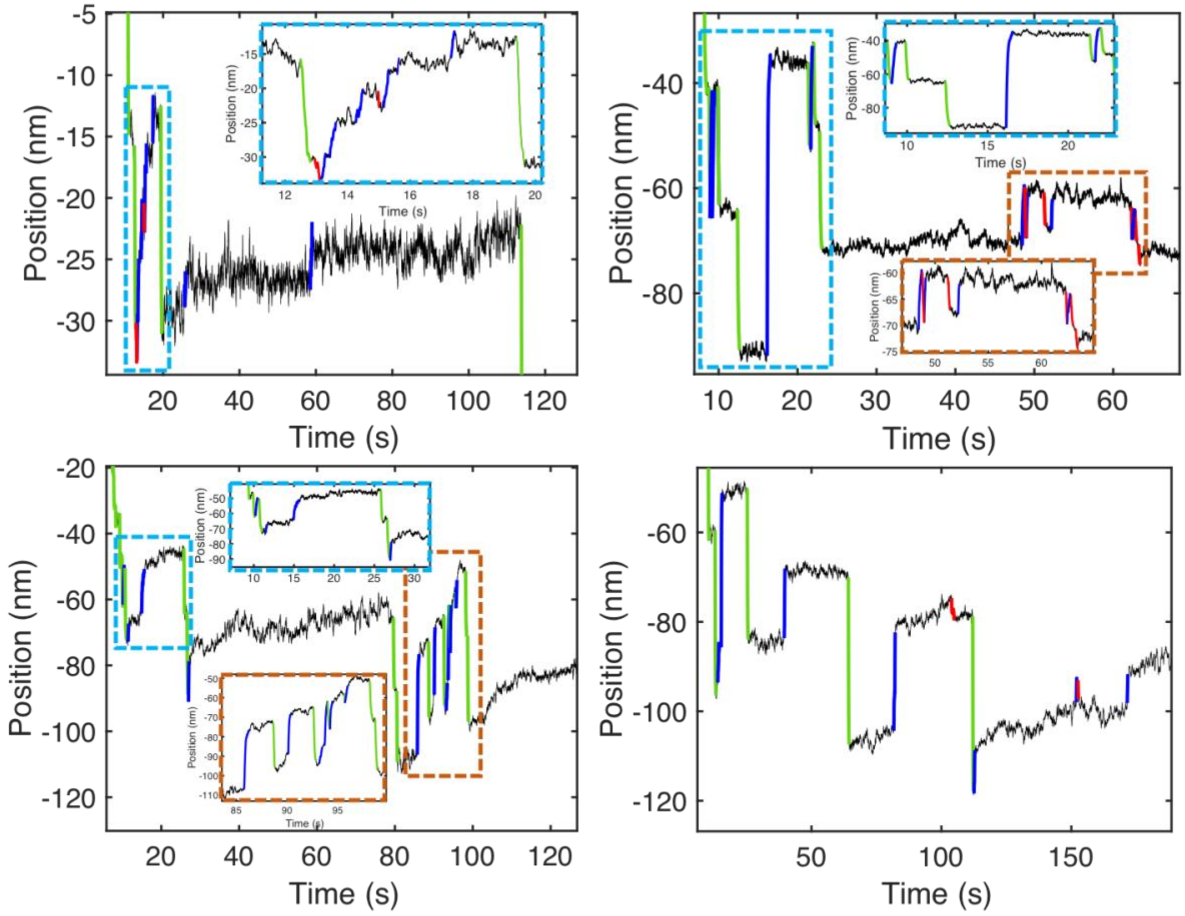

Fig. S16. Cellohexaose and microstructure. Example traces of cellulose biosynthesis in the presence of 0.45 mM cellohexaose show numerous microstructure unfolding or folding events. Stage translations are represented in green, extension events are blue, and retractions are shown in red. Cyan and orange insets display zoomed images of their respective boxed regions. In general, there were approximately three times as many microstructure events per trace in the presence of cellohexaose than in its absence (*SI Appendix* Fig. S6), further highlighting cellulose's propensity to self-associate. All data presented were recorded in the 2-8 pN range.

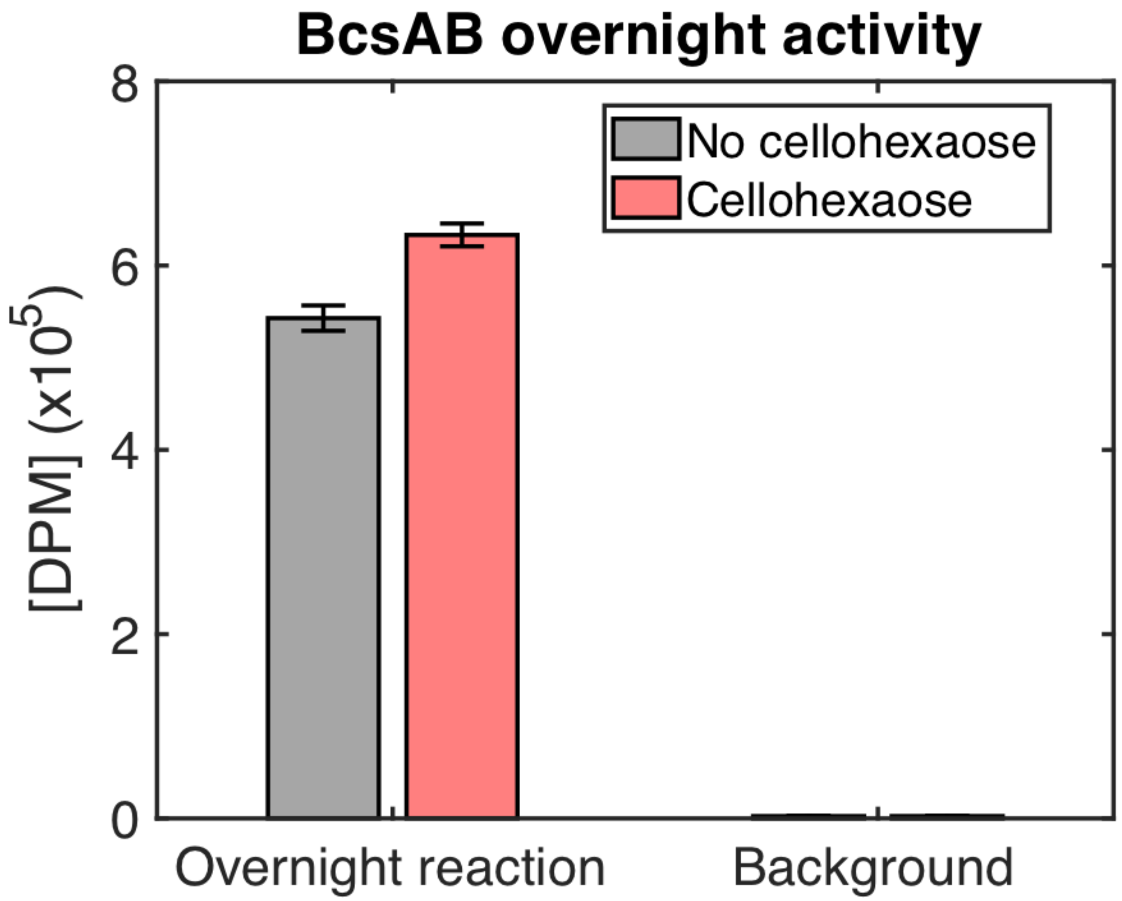

Fig. S17. BcsAB bulk overnight activity with cellohexaose. Overnight activity experiments of detergent solubilized BcsAB in the presence and absence of 0.45 mM cellohexaose. There was a significant increase in the amount of cellulose biosynthesized by BcsAB with cellohexaose present as opposed to without, indicating that hybridization aids in the catalytic reaction of cellulose elongation. Free-floating cellohexaose and extended single-strand cellulose are likely at higher energy states than when hydrophobic faces can stabilize through cellulose self-association. The energy of hybridization would then contribute to overcoming the activation energy of glucose addition. The background measurements contained no BcsAB.

Reference	Velocity	Temperature	Preparation	Species
This work	0.22 nm/s	21°C	Isolated single synthase in Nanodisc	*Rhodobacter Sphaeroides* (Bacteria)
This work	0.26 nm/s	21°C	Isolated single synthase in Nanodisc with cellohexaose	*Rhodobacter Sphaeroides* (Bacteria)
This work	1.2 nm/s	37°C	Isolated single synthase in Nanodisc	*Rhodobacter Sphaeroides* (Bacteria)
Du et al.	1.5 nm/s	30°C	Purified synthase in detergent	*Gluconacetobacter hansenii* (Bacteria)
Omadjela et al.	45 nm/s	37°C	Synthase expressed in inverted membrane vesicles	*Rhodobacter Sphaeroides* (Bacteria)
Cifuentes et al.	2 nm/s	25°C	Synthase rosettes in detergent	*Nicotiana tabacum* cv. BY-2 (Plants)
Paredez et al.	2.5-8.3 nm/s	Not reported	Synthase rosettes *in vivo*	*Arabidopsis* plants (Plants)

Table S1. Comparison of biosynthesis velocities across studies (4-7).
*This study measures synthesis by monitoring the release of UDP, which includes hydrolysis by transfer to water. Therefore, this method is not an accurate assessment of cellulose elongation.

Metric	Motility buffer without additives	Cellotetraose (5mM)	Cellotetraose (50 mM)	Cellohexaose (0.45 mM)
Persistence Length (nm ± SEM)	6.2 ± 0.4<br>N = 104	5.6 ± 0.4<br>N = 57	9.5 ± 0.4<br>N = 113	9.6 ± 0.5<br>N = 134
Axial Stiffness (pN ± SEM)	40.7 ± 2.5<br>N = 78	44.1 ± 3.0<br>N = 53	60.9 ± 3.8<br>N = 107	68.5 ± 5.4<br>N = 134
% motility traces w/ microstructure	49%	40%	45%	84%
Mean number of structure events per trace	1.5	1.4	1.14	5.1
Mean extension (retraction) size (nm ± SEM)	10.6 ± 1.9<br>N = 73<br>(4.6 ± 0.7)<br>N = 26	-	-	13.5 ± 1.0<br>N = 196<br>(6.6 ± 0.3)<br>N = 63
% stretches w/ microstructure	26%	40%	69%	94%
Mean number of structure events per stretch	0.45	0.36	0.38	1.1
Velocity (nm s^{-1} ± SEM)	0.22 ± 0.01<br>N = 176	0.19 ± 0.3<br>N = 20	0.19 ± 0.2<br>N = 24	0.26 ± 0.3<br>N = 45

Table S2. Comparison of various properties of both BcsAB and cellulose in the presence and absence of cello-oligosaccharides of different lengths. The mean number of events per trace/stretch represents the total number of events divided by the total number of traces/stretches. Mechanical properties appear unchanged in the presence 5 mM of cellotetraose. The high solubility of cellotetraose may weaken its association to cellulose compared to more hydrophobic oligosaccharides or other cellulose strands. At high concentrations (50 mM) of cellotetraose, cellulose becomes straighter and stiffer with an increase in persistence length and axial stiffness, indicating spontaneous hybridization. The concentration dependence suggests an association/dissociation constant on the order of 10 mM. Cellohexaose, near saturating conditions, was able to hybridize at a much lower concentration. The increase in mechanical properties of cellulose in the presence of cellohexaose is comparable to those seen from introducing cellotetraose. Cellohexaose's greater hydrophobicity likely decreases the association/dissociation constant and encourages binding to cellulose. Hydrophobic cello-oligosaccharides hybridizing to cellulose surprisingly increase both the frequency and size of microstructure folding/unfolding events, with the greatest increase occurring in the presence of cellohexaose. The microstructure results suggest an intense propensity for cellulose to self-associate. Motility experiments revealed an increase in velocity in the presence of cellohexaose and no change or a small decrease in productivity in the presence of cellotetraose. Seeing as cellohexaose readily hybridizes to cellulose, while cellotetraose requires much larger concentrations to achieve similar results, hydrophobic interactions likely play a role in cellulose self-association and contribute to the cellulose synthesis process in BcsAB.

SI References

1. D. Banik *et al.*, Single Molecule Force Spectroscopy Reveals Distinctions in Key Biophysical Parameters of αβ T-Cell Receptors Compared with Chimeric Antigen Receptors Directed at the Same Ligand. *J. Phys. Chem. Lett. 2021* **12**, 7573-7573 (2021).
2. Y. Shin *et al.*, Single-molecule denaturation and degradation of proteins by the AAA+ ClpXP protease. *Proceedings of the National Academy of Sciences of the United States of America* **106**, 19340-19345 (2009).
3. B. P. English *et al.*, Ever-fluctuating single enzyme molecules: Michaelis-Menten equation revisited. *Nat Chem Biol* **2**, 87-94 (2006).
4. J. Du, V. Vepachedu, S. H. Cho, M. Kumar, B. T. Nixon, Structure of the cellulose synthase complex of Gluconacetobacter hansenii at 23.4 Å resolution. *PLoS ONE*, 1-24 (2016).
5. O. Omadjela *et al.*, BcsA and BcsB form the catalytically active core of bacterial cellulose synthase sufficient for in vitro cellulose synthesis. *Proceedings of the National Academy of Sciences of the United States of America* **110**, 17856-17861 (2013).
6. C. Cifuentes, V. Bulone, A. M. C. Emons, Biosynthesis of Callose and Cellulose by Detergent Extracts of Tobacco Cell Membranes and Quantification of the Polymers Synthesized in vitro. *Journal of Integrative Plant Biology* **52**, 221-233 (2010).
7. A. R. Paredez, C. R. Somerville, D. W. Ehrhardt, Visualization of cellulose synthase demonstrates functional association with microtubules. *Science* **312**, 1491-1495 (2006).
